# Next-Generation Cancer Treatment: Photoimmunotherapy’s Promise for Unresectable Head and Neck Cancers

**DOI:** 10.3390/pharmaceutics17060716

**Published:** 2025-05-29

**Authors:** Laura Marinela Ailioaie, Constantin Ailioaie, Gerhard Litscher

**Affiliations:** 1 Department of Medical Physics, Alexandru Ioan Cuza University, 11 Carol I Boulevard, 700506 Iasi, Romania; lauraailioaie@yahoo.com (L.M.A.); laserail_mail@yahoo.com (C.A.); 2 Swiss University of Traditional Chinese Medicine, SWISS TCM UNI, High-Tech Acupuncture and Digital Chinese Medicine, 5330 Bad Zurzach, Switzerland; 3 President of the International Society for Medical Laser Applications (ISLA Transcontinental), German Vice President of the German-Chinese Research Foundation (DCFG) for TCM, Honorary President of the European Federation of Acupuncture and Moxibustion Societies, Honorary Professor of China Beijing International Acupuncture Training Center, China Academy of Chinese Medical Sciences, Honorary President of the American Association of Laser Acupuncture Therapy (ASLAT), USA, Former Head of two Research Units and the TCM Research Center at the Medical University of Graz, 8053 Graz, Austria

**Keywords:** ASP-1929, augmented reality, epidermal growth factor receptor, head and neck squamous cell carcinoma, immune checkpoint inhibitors, immune-related adverse events, IR700, mixed reality, NIR-PIT, virtual reality

## Abstract

Traditional oncological therapies have contributed to reducing the global cancer burden; however, they have not achieved complete eradication, nor have they effectively prevented relapses, minimized toxicity, or preserved immune function. Recent advances, particularly the introduction of immune checkpoint inhibitors (ICIs) and CAR-T cell therapies, have markedly improved clinical outcomes and overall survival in certain cancer subtypes. Nevertheless, response rates remain suboptimal, and adverse immunological events are frequent. This review starts by highlighting the FDA-approved ICIs currently utilized in cancer immunotherapy, emphasizing those that have demonstrated clinical efficacy in recent years. The true focus of our analysis is on the latest clinical applications of near-infrared photoimmunotherapy (NIR-PIT). This emerging modality is evaluated in patients with head and neck cancers (HNC), particularly in cases that are unresectable, locally advanced, or recurrent. Finally, the review explores the current landscape and prospects of NIR-PIT, considering its potential to enhance therapeutic efficacy and extend relapse-free survival. Photoimmunotherapy is a promising, molecularly targeted option for patients with limited prognosis, offering new hope where conventional therapies fail. By synthesizing recent clinical trial data, this work highlights how NIR-PIT could bridge the translational gap between preclinical research and clinical practice. The integration of advanced technologies and interdisciplinary collaboration among researchers, clinicians, and technologists will be critical in optimizing NIR-PIT, improving its accuracy, efficacy, and safety, and ultimately advancing standards of cancer care and patient survival.

## 1. Introduction

Cancer represents a significant public health burden worldwide, with an estimated 10 million deaths annually, making it the second leading cause of death in the US. Recent medical research has sparked new breakthroughs in diagnostics by applying artificial intelligence, sequencing the cancer genome, and developing precision cancer treatments [1,2,3,4].

Over several decades, three conventional methods of cancer therapy have been consolidated: surgery, radiotherapy, and chemotherapy, which are already included in standardized protocols and strictly followed by oncologists practicing allopathic medicine. Each technique aims to reduce the burden of cancer, while underestimating adverse effects. Experience has shown that each of these treatment methods significantly affects normal tissues, including immune system cells, which contributes to the weakening of the body with detrimental consequences in the subsequent recovery of the patient. Traditional methods of oncological treatment used for more than half a century, although they have decreased the heavy burden of cancer worldwide, have not solved the problem of complete eradication, stopping relapses, toxic side effects, and disruption of the immune system. Science has advanced, and research has brought new hope with immunotherapy, which uses cytokines to activate T lymphocytes, immune checkpoint inhibitors (ICIs), the gradual elimination of regulatory T lymphocytes (Tregs), and adoptive cell therapies (ACT) to selectively control tumor growth. In recent years, scientific evidence has emerged for these therapies, particularly for those blocking ICIs and chimeric antigen receptor transduced T (CAR-T) cell therapy, which have significantly altered clinical responses and have sustainably improved overall survival in certain subgroups of patients with specific types of cancer. However, even if the results have been spectacular in some cases, overall, the percentage of positive responses is far too low compared to toxic and immunological adverse events, the latter referred to as immune-related adverse events (irAEs). Because current cancer immunotherapies utilize the cytotoxic effect of activated immune cells to kill cancer cells, this nonspecific activation phenomenon can trigger autoimmune pathologies in normal tissue. These issues, as well as the lack of unique antigens, antigen loss in cancer cells, and the immunosuppressive tumor microenvironment (TME) of solid tumors, have prompted research to find other methods by which cancer cells can be selectively destroyed without damaging the surrounding structures. Antigen loss, i.e., loss or reduction of expression of the targeted antigen on cancer cells, triggers ineffective CAR-T cells, which is an important clinical concern in terms of relapses and limited treatment options [5,6,7,8,9,10,11,12].

This review highlights the US Food and Drug Administration (FDA)-authorized ICIs, applied today in the immunotherapy of various types of cancer, that in recent years have demonstrated efficacy. The true focus of our analysis is on the latest clinical applications of NIR-PIT. The aim was to examine the results of the most recent applications of NIR-PIT, as a cutting-edge approach to the treatment of resistant cancer, from clinical trials conducted in human head and neck cancers (HNC), especially in unresectable, locally advanced or locally recurrent ones.

Finally, the review explores the current landscape and prospects of NIR-PIT, considering its potential to enhance therapeutic efficacy and to increase the hope for relapse-free survival in cancer patients.

## 2. Brief History of PIT and Its Links to Current Immunotherapy

Observations on the benefits and efficacy of light on living organisms have facilitated the invention and development of various devices and techniques for applying phototherapy in various pathologies in animals and humans, including the treatment of cancer. Photodynamic therapy (PDT) is one of the recent methods of cancer treatment, and it is based on the ability of some photosensitizers (PSs) to absorb laser light after exposure to the appropriate wavelength and subsequently release reactive oxygen species (ROS) with a destructive effect on cancer cells, protecting neighboring structures. Currently, even if PDT resolves some cases with benefits, reduced neighboring invasion, and the possibility of repeated and precise hitting of pathological structures if necessary, being less invasive and more precise compared to other treatments such as surgery, it still has many shortcomings. Since PDT requires oxygen to release ROS, its efficacy is compromised in many cases due to tumor hypoxia. Another obstacle to PDT is represented by PSs that produce side effects of prolonged photosensitivity for several weeks or have a non-specific distribution and can lead to damage to nearby tissues. But the biggest deficiency of this innovative method is the limited penetration of light into the depth of the tumor [13,14,15,16].

Combining PDT with other treatment modalities, such as targeted therapies, gene therapies, or immunotherapy, could lead to increased survival rates, reduced relapses, and decreased side effects. Photoimmunotherapy (PIT), as a targeted type of PDT, has emerged as a promising new method, arising from the association of phototherapy and immunotherapy, which resulted in the effective and completely specific removal of the primary cancer, its metastases, and the prevention of recurrences [17].

For the first time in 1983, Mew et al. used the term “photoimmunotherapy” in an article published following experimental research on animals bearing M-1 tumors (DBA/2J M-1 myosarcoma) that they treated with targeted PDT, in which they associated intravenous (I.V.) administration of antibodies conjugated to the conventional PS hematoporphyrin, with the aim of destroying cancer cells based on the cytotoxicity of ROS resulting from this procedure on cancer cells [18].

This initial experiment brought great hopes in cancer therapy, but the results were not as expected, as well as those in subsequent attempts, because the studies were limited only to killing tumor cells in vitro, or in vivo by administering antibodies conjugated with hydrophobic PSs that accumulated in the liver. On the other hand, the cytotoxicity triggered by the release of ROS led mainly to the death of necrotic cells and produced damage to the morpho-functional structure of both targeted cells and non-targeted cells in the vicinity. Although several combinations of conventional PSs with different antibodies were further tested to improve their delivery and selectivity, these PSs were not authorized for administration in clinical practice [19,20,21,22].

When we talk about the basics of PIT, it finds its origins in preclinical experiments and then clinical applications that demonstrated that by activating the anti-cancer immune system, tumor structures can be eliminated. In fact, it departed from the experimental applications of using immunotherapy in cancers by stimulating the host immune system to recognize and then attack cancer cells. During these experiments, it was observed that the TME has a double role in the evolution of cancer; on the one hand, it stimulates the growth of the tumor by generating favorable conditions, and on the other hand, it can hinder the progression of the tumor by stopping growth or even eliminating the cancer cells. Evidence of the role of reducing immune system function and promoting self-tolerance by canceling the inflammatory activity of T lymphocytes by programmed cell death protein 1 (PD-1), expressed on the surface of activated T lymphocytes, clarified the role of this protein in the prevention of autoimmune diseases, but also in promoting the expansion of cancer cells [18,23,24,25,26,27].

It is already known that the PD-1 protein is an immune checkpoint that facilitates the programmed cell death (apoptosis) of antigen-specific T lymphocytes in the lymph nodes, but at the same time, it can decrease the apoptosis of regulatory T lymphocytes (anti-inflammatory, suppressor T lymphocytes). PD-1 works as a receptor for two ligands: programmed cell death ligand 1 (PD-L1) and programmed cell death ligand 2 (PD-L2). The immune checkpoint pathways PD-1 and cytotoxic T-lymphocyte associated antigen 4 (CTLA-4, also known as CD152) decrease T-lymphocyte activation, inducing an immunosuppressive state to maintain peripheral tolerance, but this can be taken advantage of by tumor cells that multiply, grow, and develop rapidly instead of being eliminated by the immune system. The PD-1 and CTLA-4 receptors inhibit the anti-tumor response of CD4+ and CD8+ T lymphocytes after attachment of PD-L1 and PD-L2, expressed on the surface of tumor cells. PD-L1 with an immunosuppressive role, is expressed by antigen-presenting cells, cancer cells, and other cells in the TME. The attachment of the PD-L1 to PD-1 stops the action of T lymphocytes from destroying tumor cells in the body. Stopping the interaction between the PD-1 protein and its ligand or PD-L1 with an inhibitory immune checkpoint (anti-PD-L1 or anti-PD-1) can stimulate anti-tumor activity, a phenomenon known as immune checkpoint blockade (ICB). In the case of tumor processes, T-lymphocyte regulatory pathways are often hyper-stimulated, and blocking immune checkpoints has revolutionized cancer management by maximizing the activity of anti-tumor T lymphocytes. Clinical trials with ICIs that target PD-1, PD-L1, and CTLA-4 have yielded exceptional results in recent years in certain types of neoplasia, which led experts at the FDA to approve the first ICI, the CTLA-4 inhibitor (Ipilimumab), in 2011 [28,29,30,31].

ICIs operate by activating and modulating the body’s natural immune system to defend itself against cancer. Currently, there are 14 ICIs approved by the FDA and administered conventionally for treating 20 cancer types and any type of solid tumor that shares certain molecular characteristics [32].

The FDA-approved ICIs targeting CTLA-4, PD-1, PD-L1, and lymphocyte activation gene-3 (LAG-3) are presented in Table 1 [33,34,35,36,37,38,39,40,41,42,43,44,45,46,47,48,49,50,51,52,53,54].

In the last decade, ICIs have brought great hope to patients with cancer, especially inoperable cases, but there are still many unknown and unresolved issues related to pharmacokinetics, pharmacodynamics, and the existence of specific biomarkers for high-efficiency use, and the most pressing impediments are intrinsic or tumor-acquired resistance and toxicity reactions, which limit their use in clinical practice.

Because the results of treatments with antibodies targeting CTLA-4 or PD-1/PD-L1 are still unsatisfactory, current research has shifted to studies and estimates of a new generation of ICIs, such as LAG3, T cell immunoglobulin and mucin-domain containing 3 (TIM3), and T cell immunoglobulin and ITIM domain (TIGIT) [55,56,57,58,59].

Although ICIs have widened the window for curing malignant diseases, so far, only a fraction of patients with certain forms of cancer respond to this modality of therapy. Since many patients do not respond positively or present adverse events, e.g., irAEs, such as gastrointestinal, endocrine, dermatological, allergic, or hematological toxicity, etc., the use of combinations with phytochemicals with promising anti-cancer prospects is being attempted. The advantages of phytochemicals are precisely given by the strong antitumor effect with reduced toxicity, because they eliminate cancer cells by modulating the expression of ICIs or their ligands, stimulate T lymphocytes, inhibit the cell cycle, promoting apoptosis and cooperation with the body’s defense structures, such as the intestinal microbiota and components of the innate immune system, rather than the simple and direct killing of targeted cancer cells, which can be reduced or avoided to the maximum. Most phytochemicals used in combination with conventional drugs in cancer therapy have antioxidant, anti-inflammatory, or immunoprotective properties, so they can avoid cancer cell resistance and protect normal structures from the toxic action of chemo- and radiotherapy. Currently, phytochemicals show great promise and considerable potential to improve the efficacy of anticancer treatments, including traditional therapy and recent immunotherapy [60,61,62,63].

**Table 1:** FDA-authorized immune checkpoint inhibitors [33,34,35,36,37,38,39,40,41,42,43,44,45,46,47,48,49,50,51,52,53,54].

Action/ Target	Generic Name	Chemical Formula	Brand Label	Year of Approval	Initial Treated Cancer	References
CTLA-4	Ipilimumab	C_6742_H_9972_N_1732_O_2004_S_40_	Yervoy	25 March 2011	Advanced melanoma that has metastasized or cannot be surgically removed.	Ref. [33]
CTLA-4	Tremelimumab	C_6500_H_9974_N_1726_O_2026_S_52_	Imjudo	October 2022	Coupled with Durvalumab for the treatment of hepatocellular carcinoma.	Ref. [34]
PD-1	Pembrolizumab	C_6504_H_10004_N_1716_O_2036_S_46_	Keytruda	4 September 2014	Metastatic malignant melanoma.	Ref. [35]
PD-1	Nivolumab	C_6362_H_9862_N_1712_O_1995_S_42_	Opdivo	22 December 2014	Therapeutic area: Melanoma; Non-small cell lung cancer; Small cell lung cancer; Malignant pleural mesothelioma; Renal cell carcinoma; Hodgkin lymphoma; Squamous cell carcinoma (SCC) of the head and neck; Urothelial carcinoma; Colorectal cancer (MSI-H or dMMR); Hepatocellular carcinoma; Esophageal squamous cell carcinoma (ESCC); Gastric cancer; Esophageal adenocarcinoma.	Ref. [36]
PD-1	Cemiplimab	C_6380_H_9808_N_1688_O_2000_S_44_	Libtayo	28 September 2018	Treatment of advanced cutaneous squamous cell carcinoma (CSCC).	Ref. [37]
PD-1	Dostarlimab	C_6420_H_9832_N_1680_O_2014_S_44_	Jemperli	April 2021	Accelerated approval for the treatment of recurrent or advanced mismatch repair deficient (dMMR) endometrial cancer that is progressing despite treatment with platinum-containing chemotherapy regimens.	Ref. [38]
PD-1	Retifanlimab	C_6456_H_9934_N_1702_O_2032_S_46_	Zynyz	March 2023	Accelerated approval for metastatic or recurrent locally advanced Merkel cell carcinoma (MCC).	Ref. [39]
PD-1	Toripalimab	C_6548_H_10104_N_1728_O_2054_S_44_	Loqtorzi	October 2023	Treatment of selected patients with nasopharyngeal carcinomas.	Ref. [40] Ref. [41]
PD-1	Tislelizumab	C_6410_H_9916_N_1686_O_2009_S_40_	Tevimbra^®^ (tislelizumab-jsgr).	4 March 2025	In combination with platinum-containing chemotherapy, for the first-line treatment of adults with unresectable or metastatic ESCC, whose tumors express PD-L1 (≥1). The structure of tislelizumab has been modified to maximally inhibit the binding of PD-1 to programmed death ligand 1 (PD-L1).	Ref. [42] Ref. [43] Ref. [44]
PD-L1	Atezolizumab	C_6446_H_9902_N_1706_O_1998_S_42_	Tecentriq, Tecentriq Hybreza	18 October 2016	Patients with tumors that express PD-L1 and cannot receive platinum-based chemotherapy or do not respond to this therapy. In November 2022, the manufacturer (Genentech) voluntarily withdrew the use of Atezolizumab only for the urothelial carcinoma. Rest of indications remain unaffected: Non-small cell lung cancer; Breast cancer (PD-L1 expressed, HR negative, HER2 negative); Small cell lung cancer; Hepatocellular carcinoma.	Ref. [45] Ref. [46]
PD-L1	Avelumab	C_6374_H_9898_N_1694_O_2010_S_44_	Bavencio	23 March 2017	Merkel cell carcinoma, metastatic urothelial carcinoma, or renal cell carcinoma.	Ref. [47]
PD-L1	Durvalumab	C_6502_H_10018_N_1742_O_2024_S_42_	Imfinzi	27 March 2020	Combined with Etoposide and either Carboplatin or Cisplatin as first-line treatment for extensive-stage small cell lung cancer (ES-SCLC).	Ref. [48]
PD-L1	Cosibelimab; Cosibelimab-ipdl;	C_6388_H_9912_N_1716_O_2032_S_44_	Unloxcyt	13 December 2024	For patients with metastatic cutaneous squamous cell carcinoma (mCSCC) or locally advanced CSCC (laCSCC) who are not candidates for curative surgery or radiation.	Ref. [49] Ref. [50]
LAG-3	Nivolumab and Relatlimab; Nivolumab and Relatlimab-rmbw.	C_6462_H_9990_N_1714_O_2074_S_42_ and C_6584_H_10106_N_1718_O_2102_S_38_	Opdualag	March 2022	Nivolumab and Relatlimab (Opdualag) is indicated as first-line treatment of advanced (unresectable or metastatic) melanoma in people aged 12 years and older.	Ref. [51] Ref. [52] Ref. [53] Ref. [54]

However, we have to say that studies have revealed that phytochemicals currently administered for various combinations in cancer therapy still have many deficiencies, because most of the time, their concentrations are insufficient for cancer prevention or therapy, and on the other hand, their low solubility in water, poor stability, in vivo short half-life, poor digestive absorption, rapid metabolism, oral bioavailability, and low functionality prevent these substances from exerting their desired anticancer functions, even though today we have drug delivery systems (DDSs) based on nanomaterials. At the same time, it must be said that not all phytochemicals are free from toxicity, because in some cases, we have to use high doses to reach the effective clinical dose, which predisposes to allergic or toxic renal or hepatic adverse reactions, sometimes severe. Since most phytochemicals have been shown to inhibit tumor expansion by reducing PD-L1 expression, some phytochemicals work in reverse by increasing PD-L1 levels, which will make them less effective in various types of tumors [64,65,66].

Although the results of single immunotherapy and, in some cases, combining it with phytochemicals have been spectacular, the overall response rate is still low, mainly due to the lack or deficiency of tumor T-cell infiltration, which characterizes the so-called “cold tumors” [67].

Added to these are irAEs detected in up to 90% of patients treated with a CTLA-4, and in 70% of those treated with PD-1/PD-L1 checkpoint inhibitors [68].

## 3. Basics of Near-Infrared Photoimmunotherapy and Clinical Studies

### 3.1. Fundamental Mechanisms of Near-Infrared Photoimmunotherapy

Near-infrared photoimmunotherapy (NIR-PIT) is a new modality for current molecularly targeted cancer therapy applied by administering an antibody (Ab) conjugated to the hydrophilic silicon-phthalocyanine derivative photoabsorbing dye, the IRDye 700DX (IR700) platform, followed by irradiation with light in NIR (680–800 nm) that can selectively kill cancer or immune regulatory cells and then induce host immune responses. The fluorescent dye IRDye operates at an average absorption and emission wavelength in the NIR spectrum of 700 nm (IRDye 700DX). After local exposure to NIR light, a photo-induced ligand release reaction occurs, which degrades the target cell and actively triggers immunogenic cell death (ICD) of cancer cells with minimal or no side effects on adjacent normal cells. Furthermore, NIR-PIT can trigger an immune response at the level of metastases and stop the spread of cancer [69,70,71,72].

Rakuten Medical, Inc., a biotechnology company that owns the Alluminox™ platform, develops and commercializes biologics for the selective and precise targeting of cancer cells and the induction of tumor necrosis. The company has offices in Japan, Taiwan, Switzerland, India, and the United States, where it is headquartered. Rakuten Medical, Inc. is commercializing Cetuximab Sarotalocan, also known as ASP-1929 (RM-1929) or cetuximab-IRDye 700DX conjugate, which is an anti-[EGFR (epidermal growth factor receptor), avian erythroblastic leukemia viral oncogene homolog], chimeric monoclonal antibody (mAb) conjugated covalently to IRDye 700DX (cet-IR700) near-infrared photosensitizing dye [73,74], that received accelerated approval from the FDA in January 2018 for the treatment of recurrent head and neck cancer (rHNC). In September 2020, the Japan Pharmaceutical and Medical Device Agency approved antibody–photoabsorber conjugate (APC) ASP-1929 (Japanese brand name: Akalux^®^ 250 mg for intravenous infusion) as the first NIR-PIT drug targeting the EGFR and, at the same time, a diode laser device (BioBlade™, Rakuten Medical Inc., Tokyo, Japan) for use in clinical practice [75,76,77].

EGFR is the most common antigen expressed on the surface of cancer cells, so EGFR-targeted NIR-PIT is now being successfully used for various types of tumors that express this antigen. For patients whose cancer does not express the EGFR antigen, it is necessary to find another structure that NIR-PIT can specifically target. There are particular concerns about improving the results of NIR-PIT by developing new targeting molecules, delivering NIR intravenously via catheter to the exact area of interest, and monitoring the cancer in real time. NIR-PIT targeting the EGFR in invasive head and neck tumors is currently approved for clinical use in Japan, and a phase III clinical trial is underway worldwide [17,74,76].

NIR-PIT is recommended as a procedure that overcomes these challenges, as well as those of conventional PDT or photothermal therapy (PTT), which work through the effect of cytotoxic singlet oxygen (1O2) release and hyperthermia, respectively [78].

Molecularly targeted NIR-PIT is supported by the systemic injection of an mAb conjugated with a silicon-phthalocyanine derivative, which is water-soluble and absorbs in the NIR, i.e., IRDye 700DX (IR700). After the mAb selectively binds to the antigens on the surface of the cancer cells, NIR irradiation will lead to photochemical reactions of the APC, which will trigger the rupture of the membrane and cause cell death. NIR-PIT activates the immune response by inducing ICD. This selective ICD process induced by NIR-PIT can reawaken multiple tumor-specific immune responses, through the immune initiation of local dendritic cells (DCs) and the maturation and proliferation of naïve cancer-specific T lymphocytes. NIR-PIT is able to vigorously control immunosuppressive cells in the TME, including Tregs lymphocytes and myeloid-derived suppressor cells (MDSCs), adjusting the normal functioning of the immune system and overcoming the obstacle of conventional immunological drugs [79,80].

**Figure 1:**
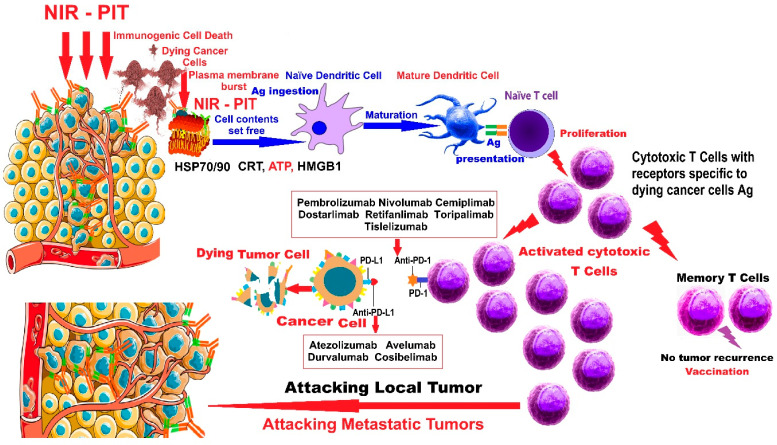
Action of NIR-PIT combined with ICIs in malignant tumors. (Figure 1 was imagined and drawn by L.M.A. using Microsoft Paint 3D for Windows 10 and completely free illustrated material from SeekPNG.com (accessed on 26 April 2025), for which we are very grateful; and includes elements from Figure 4 [16] already published by L.M.A., as first author).

NIR-PIT actively triggers immunogenic cell death of cancer cells; it initiates host immunity against target cancer cells with the fewest or no adverse effects on neighboring normal cells. NIR-PIT simultaneously activates the immune system against multiple antigens released from dying cancer cells, resulting in a more comprehensive response through a broader spectrum of antigens specific to the targeted tumor. The remote immune response generated by NIR-PIT can stop the dissemination of tumor cells (Figure 1).

The proposed mechanism of action for NIR-PIT combined with ICIs in malignant tumors, presented in Figure 1, is as follows: NIR-PIT results in disruption of cancer cell membranes, which release signals and molecules such as heat shock proteins 70 (Hsp70) and 90 (Hsp90), calreticulin (CRT), adenosine triphosphate (ATP), and high-mobility group box 1 (HMGB1) protein, activating naϊve dendritic cells in the vicinity and in the TME, which will mature by ingesting cancer-specific antigens (Ags) freshly released by dying tumor cells. The mature DCs will stimulate and instruct naϊve T cells to develop into cytotoxic T cells with receptors specific to the antigens of dying cancer cells. The freshly activated cytotoxic T cells will multiply and attack local and metastatic tumors, triggering a stimulated memory anticancer immune response. “Vaccination” means that NIR-PIT induces immune memory against cancer, like a cancer vaccine. Although NIR-PIT acts locally, an induced systemic activation of the immune system occurs, and by combining NIR-PIT with ICIs, as adjuvant therapy, a much stronger immune response can be achieved.

Among other advantages, NIR-PIT will induce the rapid and extensive death of cancer cells participating in the enhanced permeability and retention (EPR) effect, a phenomenon known as super-enhanced permeability and retention (SUPR), which translates into increased delivery volume of other antibodies, APC, and nanometric drugs or even macromolecules. Through the arguments brought by its potential effectiveness and safety, NIR-PIT constitutes a treatment alternative with wide perspectives in different types of cancer and other non-cancerous conditions (infections, allergies, pain, metabolic diseases, autoimmune and tissue engineering) [81,82,83].

Compared to conventional therapy, NIR-PIT has great superiority, but despite this, there are still many problems that remain to be solved, including insufficient penetration of NIR light into large tumors where the efficiency is lower and there is a risk of lymph node metastases; modest results in some early-stage cancers that express multiple tumor-specific antigens; and, last but not least, the fact that most in vivo studies are experimental in animal models. Consequently, further investigations are still needed to understand the mechanisms of eliminating metastases, preventing tumor recurrence, and establishing the criteria for selecting ideal patients and the benefits of treatments associated with NIR-PIT [22,84,85].

### 3.2. Applications of NIR-PIT in Clinical Trials on Human Head and Neck Cancers

Head and neck cancer is one of the most common types of cancer, ranking seventh worldwide, with 946,456 new cases/year and 482,001 deaths reported in 2022, according to GLOBOCAN. The incidence of this type of malignant cancer will grow by 40% by 2040 and may reach 600,000 new cases annually [86,87,88].

The current alarming trend of increasing incidence can be largely attributed to preventable risk factors such as smoking, alcohol consumption, and sexually transmitted Human Papilloma Virus (HPV) infections. The incidence of this type of cancer is increasing even in developed countries with decreasing smoking rates. Almost 90% of all HNCs are SCCs hat originate in the epithelium of the oral cavity, pharynx, and larynx, and the remainder are cancers of the nasal cavity and salivary glands [87,89,90].

In 2024, according to the EpiCast report for HNC in the seven major markets (7MM: US, France, Germany, Italy, Spain, UK, and Japan), the United States was expected to have an increase in the number of incident cases diagnosed as HNC to 65,663, compared to the United Kingdom, where the number of incident cases diagnosed with HNC was lower, around 12,702 [91,92,93].

Currently, the treatment of recurrent head and neck squamous cell carcinoma (rHNSCC) is typically accomplished with platinum-based chemotherapeutic agents (Cisplatin, Carboplatin) and targeted therapies with EGFR inhibitors, such as Cetuximab (Erbitux) and Panitumumab (Vectibix); however, the response to these agents is limited because neoplastic cells can become resistant due to a mutation or find a new way to grow, so that the treatment no longer works. For this reason, targeted therapies may work best in combination. Immune checkpoint inhibitors have brought great hope, but response rates for rHNSCC are modest. Given these problems, there is a pressing need to provide alternative therapeutic methods for the control of patients with rHNSCC [94,95].

The development of NIR-PIT initiated a new option, emerging separately from PIT. Good results were generated by IR700, conjugated to specific mAbs targeting the EGFR molecule on the cancer cell surface and followed by the administration of NIR radiation. This time, the effects were remarkable, achieving deep tissue penetration, leading to immediate cell death, tumor volume reduction, and ultimately eradication of subcutaneous xenograft formations in A431 (HER1 positive) and 3T3/HER2 (HER1 negative) tumor models in mice after a single dose of NIR, without cytotoxicity to the surrounding healthy tissue. This study paved the way for the use of the mAb-IR700 conjugate in human cancer therapy with NIR-PIT, targeting neoplastic cells bearing the EGFR antigen [23].

In this subchapter, we present all the clinical trials conducted and registered on ClinicalTrials.gov, summarized below in Table 2 on NIR-PIT in recurrent head and neck cancers.

In 2015, the first phase I/IIa study was initiated. In this study, patients with unresectable rHNSCC received treatment with the conjugate called RM-1929, anti-EGFR–IR700 dye conjugate followed by NIR-PIT. The study is identified in ClinicalTrials.gov as NCT02422979 (https://clinicaltrials.gov/study/NCT02422979, accessed on 14 February 2025; see also Table 2, [96]), and the first results were published in a book of abstracts and communicated at the 42nd European Society for Medical Oncology (ESMO) Congress in 2017, on nine patients with rHNSCC treated in the USA, in research funded by Aspyrian Therapeutics under the jurisdiction of the FDA. This phase 1 study was testing doses of RM-1929 and NIR-PIT on a type of malignant cancer with no hope of resolution and proved the safety and good tolerability of RM-1929 and NIR-PIT therapy with obvious clinical improvement [97].

According to reference data from the National Library of Medicine (NLM), the National Center for Biotechnology Information (NCBI), and the ClinicalTrials.gov platform (https://clinicaltrials.gov/) in the USA, six international clinical trials are posted on NIR-PIT combined with RM-1929/ASP-1929 applied in the therapy of HNC, HNSCC, rHNSCC, metastatic HNSCC, laCSCC, and mCSCC (Table 2).

In 2018 at the American Society of Clinical Oncology (ASCO) Annual Meeting I, Gillenwater et al. (2018) presented a poster published as abstract no. 6039 on the results of a phase 2a study in 30 patients with rHNSCC who received intravenous RM-1929 followed 24 h later by NIR-PIT activation applied superficially and deep intratumorally with an optical fiber. There were no local or systemic adverse reactions. Clinical parameters were significantly improved compared to standard therapy, and the median overall survival for this group in the NCT02422979 study was 278 days [98].

Cognetti et al. (2019) presented a partial phase 2a, multicenter, open-label study in 30 patients with rHNSCC who received RM-1929 by I.V. infusion followed by 24 h of local and interstitial nonthermal red light (690 nm) deep within the tumor. This research is part of clinical trial no. NCT02422979 and was presented at the 2019 American Society of Clinical Oncology Annual Meeting, 31 May–4 June 2019, Chicago, IL, USA, and published in abstract no. 6014. Results show that there were no toxicities, clinical response rates were good, and adverse reactions were mild to moderate grade 1 [99].

The final results of clinical trial no. NCT02422979 were published by Cognetti et al. (2021), who participated in the first research in the world on PIT and the drug RM-1929 administered to patients with rHNSCC with limited therapeutic options and poor prognosis. The first part of the study included nine patients and aimed to investigate the safe dose of the drug RM-1929 at a fixed dose of laser light. The initial dose of RM-1929 was 160 mg/m^2^, then increased to 320, and finally to 640 mg/m^2^. In Part II, 31 patients were enrolled between June 2015 and December 2017 and received RM-1929 at a dose of 640 mg/m^2^, and the laser fluence was 50 J/cm^2^ applied to superficial tumors and 100 J/cm delivered interstitially via fiber deep into the tumor. The results of the study demonstrate that RM-1929 photoimmunotherapy has the potential to provide breakthrough benefits for patients who have previously failed multi-agent therapies, and it is expected that this new treatment modality will offer a chance for good locoregional control in rHNSCC [100].

A new study with the ID: ASP-1929-301, NCT ID: NCT03769506 and the official title: “A Phase 3, Randomized, Double-Arm, Open-Label, Controlled Trial of ASP-1929 Photoimmunotherapy Versus Physician’s Choice Standard of Care for the Treatment of Locoregional, Recurrent Head and Neck Squamous Cell Carcinoma in Patients Who Have Failed or Progressed On or After at Least Two Lines of Therapy, of Which at Least One Line Must Be Systemic Therapy” is recruiting patients https://clinicaltrials.gov/study/NCT03769506, accessed on 14 February 2025. The estimated completion date of this study is December 2025 [101].

Patients with advanced HNSCC have a high rate of locoregional recurrence and a reduced overall survival. Despite all the therapeutic methods based on surgery, radiotherapy, aggressive chemotherapy, and immunotherapy, recurrence cannot be controlled. PIT associated with ASP-1929, which targets EGFR expressed on the surface of HNSCC tumor cells, has proven its effectiveness in rapidly destroying tumor tissue in preclinical and recent clinical studies. The results of preclinical studies of the association of anti-PD-1 drugs with PIT offer an additional chance and great hope in resolving the locoregional recurrence. Another study, identified on ClinicalTrials.gov with the ID NCT04305795, sponsored by Rakuten Medical, Inc., with the official title: “An Open-label Study Using ASP-1929 Photoimmunotherapy in Combination with Anti-PD1 Therapy in EGFR Expressing Advanced Solid Tumors”, is active and has an estimated completion date of June 2027 [102].

Cognetti et al. (2024) published updated preliminary results from this open-label phase 1b/2 study (ASP-1929-181; NCT04305795) in patients with recurrent locally advanced (rLA) and/or metastatic head and neck squamous cell carcinoma mHNSCC on the safety and efficacy of ASP-1929 PIT in combination with an anti-PD-1 drug, pembrolizumab, which was previously presented at the 2023 American Head and Neck Society Annual Meeting. The treatment protocol included ASP-1929 infusion and PIT illumination 24 ± 4 h later, to which the anti-PD-1 drug pembrolizumab was added on days 1 and 22 for a period of 6 weeks up to 24 months. Safety/tolerability, objective response rate, overall survival, progression-free survival, and duration of response were monitored. Nineteen recurrent/metastatic (r/m) HNSCC patients were enrolled, of whom 18 received both treatments. Significant adverse events included dysphagia (10.5%) and tongue edema (10.5%), and grade 4 events were laryngeal edema after PIT and tumor hemorrhage due to advanced disease; all patients survived. The results of ASP-1929 PIT therapy in combination with anti-PD-1 are promising for patients with rLA and/or m HNSCC without LR treatment options, in terms of overall survival and tolerability [103].

Three other studies, identified on ClinicalTrials.gov with the ID NCT05220748, ID NCT05265013, and ID NCT05182866, are presented and discussed below and included in Table 2.

Study no. NCT05220748, a phase 1a/1b, open-label, RM-1995 drug-dose escalation trial sponsored by Rakuten Medical, Inc., began on 24 March 2022 and was designed to enroll an estimated 36 patients with CSCC or HNSCC that had progressed despite all available standard therapies. The main objectives were to evaluate the safety, tolerability, pharmacokinetics, pharmacodynamics, and preliminary efficacy of RM-1995 as monotherapy (phase 1a) in PIT treatment or combined with pembrolizumab (phase 1b). The total number of patients was to be distributed into six cohorts and receive anti-CD25 antibody, conjugated to IRDye 700DX, followed by illumination with non-thermal red light as monotherapy (phase 1a), or in combination with pembrolizumab (phase 1b). However, following a strategic reassessment on 19 April 2023, the decision was made to withdraw this study [104].

Study no. NCT05265013, titled “A Phase 2 Single-arm Study of ASP-1929 Photoimmunotherapy Combined with Pembrolizumab in Patients with Locoregional Recurrent Squamous Cell Carcinoma of the Head and Neck, with or without Metastases, Not Amenable to Curative Local Treatment”, was sponsored also by Rakuten Medical, Inc. The aim was to investigate the effect of ASP-1929 photoimmunotherapy combined with pembrolizumab in a single arm that was to include 33 patients. Patients should receive the biological agent pembrolizumab at a dose of 200 mg every 3 weeks on days 1 and 22 of each 6-week cycle, as a 30 min I.V. infusion, combined with ASP-1929 at a dose of 640 mg/m^2^ as an I.V. infusion, administered on day 8 of each cycle, followed one day (24 h) after the end of the infusion by illumination with PIT690 Laser System on day 9 at a dose of 50 J/cm^2^ for superficial lesions and 100 J/cm for deep interstitial lesions. Each treatment cycle, which was driven by the frequency of ASP-1929 PIT administration, would last 42 days. Patients were to be treated with ASP-1929 PIT and pembrolizumab for up to 12 months, with a maximum of eight treatment cycles. The study was completed on 16 October 2024, with 16 enrolled patients. The results are not yet public [105].

Study No. NCT05182866, titled: “Phase 2, Open-label, Single-arm, Window of Opportunity Study of ASP-1929 Photoimmunotherapy with Fluorescence Imaging in Patients with Operable Primary or Recurrent HNSCC”, is also sponsored by Rakuten Medical, Inc. and began on 21 January 2022. It is an experimental study with 22 patients, who will receive treatment with ASP-1929 at a dose of 640 mg/m^2^ by I.V. infusion, followed approximately 24 h later by PIT illumination of the tumor(s) using the PIT690 Laser System with a wavelength of 690 nm at a dose of 50 J/cm^2^ for superficial lesions and 100J/cm for deep interstitial lesions. All patients will receive a combination of ASP-1929 and PIT. During illumination, the fluorescence of the IR700 component of ASP-1929 will be imaged at 830 nm with a Shimadzu Fluorescence Imaging System camera. Patients will undergo standard care surgery with or without chemotherapy or radiation, approximately 21 days after ASP-1929 PIT treatment. The study is expected to be completed on 30 June 2026 [106].

**Table 2:** Clinical trials on NIR-PIT in head and neck cancers [96,97,98,99,100,101,102,103,104,105,106].

References	No. Study	Study Start/Completion (year)	Phase	No. Patients	Treatments	Types of Cancers	Results
Ref. [96]	NCT02422979	2015-06 2019-02-25	1/2a	39	PIT + RM-1929	Recurrent Head and Neck Cancer (rHNC)	Well-tolerated therapy with valuable clinical response.
Ref. [97]	NCT02422979	2015–2017	1	9	PIT + RM-1929	rHNC	NIR-PIT with well-tolerated RM1929 and improved clinical data
Ref. [98]	NCT02422979	2015–2017	2a	30	RM-1929 + PIT superficially and deep intratumorally	rHNC	No local or systemic adverse reactions and clinical parameters were significantly improved.
Ref. [99]	NCT02422979	2019	2a	30	RM-1929 + PIT superficially and deep intratumorally	rHNC	RM-1929—PIT was well tolerated, with most AEs being mild to moderate in severity.
Ref. [100]	NCT02422979	2015-06 2019-02-25	1/2a	39	PIT + RM-1929	rHNC	Well-tolerated therapy with valuable clinical response
Ref. [101]	NCT03769506	2019-05-09 2025-12 (estimated study completion)	3	135	PIT + RM-1929 + drug chosen by the physician: docetaxel, cetuximab, methotrexate, paclitaxel	rHNC	Active study
Ref. [102]	NCT04305795	2020-12-21 2027-06 (estimated study completion)	1 and 2	23	PIT + ASP-1929 + combination with anti-PD1 therapy	rHNSCC or (m)HNSCC or laCSCC mCSCC	Active study
Ref. [103]	NCT04305795	2023	1b/2	18	PIT + ASP-1929 + combination with anti-PD1 therapy	rHNSCC or (m)HNSCC or laCSCC or mCSCC	Preliminary data. Well tolerated therapy; promising survival rates.
Ref. [104]	NCT05220748	2022-03-24 2023-01-30	1	0	RM-1995 + biological Pembrolizumab	CSCC or HNSCC	withdrawn
Ref. [105]	NCT05265013	2022-04-19 2024-03-29	2	16	PIT + ASP-1929 + biological Pembrolizumab	HNC	Study Completed
Ref. [106]	NCT05182866	2022-01-21 2026-06-30 (estimated study completion)	2	9	PIT + ASP-1929	HNC and HNSCC	Active

### 3.3. NIR-PIT in Unresectable, Locally Advanced or Locally Recurrent Head and Neck Cancers—Case Series

In this subchapter, we introduce a series of clinical cases (discussed below and presented in Table 3) treated with NIR-PIT combined or not with ICIs, monitored by different imaging techniques.

For the first time, the use of NIR-PIT in rHNSCC was conditionally approved in Japan in September 2020. The effects also included immune responses on not directly treated lesions located at a distance. NIR-PIT with cetuximab-IR700 (or AlluminoxTM) has been applied in routine clinical use since January 2021 in Japan for unresectable locally advanced or locally recurrent HNC in patients previously treated with radiotherapy. Since 2022, NIR-PIT is indicated in “unresectable, locally advanced, or locally recurrent head and neck cancer, with priority given to standard treatments such as chemotherapy, when available”. In recurrent nasopharyngeal carcinoma, NIR-PIT is a first-choice option because radical resection surgery is very complex and difficult due to the anatomical architecture [76,107,108].

Tahara et al. (2021) undertook a single-arm, open-label, phase I study to investigate the safety, preliminary efficacy, pharmacokinetics, and immunogenicity after a single cycle of PIT with RM-1929 in Japanese patients with rHNSCC. Three Japanese patients who had failed ≥3 lines of treatment, including radiotherapy, chemotherapy, cetuximab, and immunotherapy, were enrolled in the study. Treatment was performed with RM-1929 at a dose of 640 mg/m^2^ and PIT with 50 J/cm^2^ for superficial illumination, and 100 J/cm fiber diffuser length for interstitial illumination, in a single dose, applied to the submental area, the right superficial cervical nodule, the external auditory canal, and the oropharyngeal lesion. NIR-PIT with RM-1929 showed a significant clinical response with a good safety profile in all three of these heavily pre-treated patients (Table 3). The study needs to be expanded and is part of the Japanese clinical trial registry as No. jRCT2031200133 [109].

#### 3.3.1. PIT in Locally Recurrent Nasopharyngeal Carcinoma

Japanese authors Kushihashi et al. (2022) reported a case of locally recurrent nasopharyngeal carcinoma treated with endonasal photoimmunotherapy using a laser device (BioBlade^®^ Laser System; Rakuten Medical) and a cylindrical diffuser (“BioBlade SideFire Diffuser™”; Rakuten Medical, Tokyo, Japan), together with Cetuximab sarotalocan sodium at a dose of 640 mg/m^2^ administered intravenously. This case was about a 57-year-old patient who was known to have had metastatic nasopharyngeal carcinoma to the right cervical lymph node, for which he had received radiotherapy, chemotherapy, and radical neck dissection with subsequent complete response. The patient returned to the otolaryngologist after 21 years with suspected rhinosinusitis. Endoscopic examination, assisted by computed tomography (CT), magnetic resonance imaging (MRI), and positron emission tomography (PET), detected a tumor around the right Eustachian tube. Histopathological examination reveals a non-keratinizing SCC, thus a local recurrence of the initial nasopharyngeal carcinoma (SCC, rT1N0M0 Stage I). Since surgical resection was not recommended, PIT was decided upon. Since the tumor was in an awkward position and the operating space was limited, the inferior turbinate was resected, then, after perforating the tumor at the level of the lateral wall of the transnasal nasopharynx with a cylindrical diffuser, it was penetrated to a depth of at least 10 mm, and the intratumoral irradiation was performed.

Six months after the intervention, the endoscopic aspects and MRI imaging were fine, with a complete response. This case is the first in the world in which a complete response was achieved after PIT irradiation using a cylindrical diffuser for local recurrence of nasopharyngeal carcinoma. PIT opens new perspectives in the therapy of local recurrence of nasopharyngeal cancer that is difficult to surgically resect [110].

NIR-PIT is a treatment method that can destroy tumors through the immunogenic cell death phenomenon and initiate the host immune system response against the tumor [111,112,113].

#### 3.3.2. Preventive Tracheostomy Prior to NIR-PIT for Oropharyngeal Cancer

Nishikawa et al. (2022) reported two cases of patients with oropharyngeal neoplasia in whom surgery was considered high risk, for which reason NIR-PIT was administered with good results, without severe adverse events or functional disorders. The first case, an 80-year-old man, had 40 years ago received partial tongue resection surgery and radiotherapy for mandibular gingival carcinoma, which recurred in the lateral wall of the oropharynx. After a detailed examination, the tumor was considered unresectable, and therefore NIR-PIT therapy was decided. On the first day, RM-1929 and NIR-PIT illumination were administered without problems. On the second day, a tracheostomy and illumination with 20 mm long cylindrical diffusers were performed; although the tumor had shrunk, a biopsy revealed residual tumor cells, which is why a second round of NIR-PIT was performed with an increased number of diffusers. After the second cycle, tumor reduction was observed again, but MRI suspected residual tumor, for which a third round of NIR-PIT was recommended; however, the patient refused, and then, 5 months after the second cycle, transoral resection was performed, and the histopathological examination showed a different appearance from the original maxillary gingival carcinoma. Sixteen months after the operation, no recurrence occurred, and the patient remained under observation. This first case responded well, without serious adverse reactions or functional disorders. The second patient was a 77-year-old man diagnosed 6 years ago with hypopharyngeal cancer, for which he received chemo-radiotherapy and transoral resection for cancer of the base of the tongue and resection of cancer of the soft palate 1 year ago. Then, a tongue base cancer was discovered with a size < 20 mm in diameter, but considering the previous surgical history, it was considered to be high risk, and NIR-PIT was proposed. Initially, the patient received intravenous RM-1929 without complications. On the second day, tracheostomy was performed followed by laser illumination with cylindrical diffusers of 20 mm in length under endoscopy and ultrasonography. Eleven months after PIT, the mucosal surface was slightly irregular, but biopsy showed no residual cancer cells [114].

PIT administered after infusion of Cetuximab sarotalocan sodium in HNC therapy releases the photochemical “death” switch that induces irreversible disruption within 1 min in cells expressing the targeted antigen on their surface and dissipates the tumor, while receptor-negative cells in the vicinity of the tumor process remain completely unharmed. NIR-PIT can target any of the antigens expressed on the surface of the cancer cell, including CD44 and CD133 as positive markers of cancer stem cells [70,115].

Okamoto et al. (2022) reported a case of laryngeal SCC in a 76-year-old patient in whom multiple surgeries and three sets of radiotherapy were performed, but the disease had recurred and metastasized to the cervical lymph nodes, for which PIT was attempted. Cetuximab sarotalocan sodium (640 mg/m^2^) was initially administered by IV for 2 h. Laser illumination was performed locally and deeply interstitially with a cylindrical diffuser emitting a laser beam with a radius of 10 mm for a duration of 8 min and 20 s. An acne-like rash had been reported. After 6 weeks, the second session of PIT was administered with an irradiation duration of 9 min and 43 s, but the cervical CT scan revealed a 16 mm nodal metastasis in the right submandibular region and left retropharyngeal lymph node. PIT response was considered partial after the first PIT session, and the progressive evolution of the disease was observed after the second session [116].

Okamoto et al. (2022) reported another case study of a 70-year-old patient known to have SCC of the gingival area of the left jaw for which he underwent surgery, radiotherapy, chemotherapy with cisplatin, and after 6 months, due to relapse, received nivolumab. Because the tumor relapsed on the posterior wall of the maxillary sinus and extended from the periphery of the lateral pterygoid muscle to the base of the skull, a reconstructed area, PIT (690 nm red light) was decided. Cetuximab sarotalocan sodium (640 mg/m^2^) was administered as an IV infusion the day before. After induction of general anesthesia, the direction of deep infiltration of the lateral pterygoid muscle and the absence of the nearby internal carotid artery were confirmed using the navigation system pointer (Fusion™ ENT Navigation System (Medtronic, Sunnyvale, CA, USA) with magnetic field). A 50 mm needle catheter was inserted in the same direction as the pointer. A 30 mm cylindrical diffuser was inserted into the needle catheter, and the first irradiation was performed. Superficial lesions were treated with a frontal diffuser set at 38 mm. The total PIT irradiation time was 9 min and 43 s, and the total surgical time was 55 min until the end of surgery. At 3-month follow-up, the patient was considered to have had a complete response, as the superficial lesion was no longer present on endoscopy and CT, and the injury in the lateral pterygoid muscle had improved significantly. The good results of PIT without adverse effects were due to the navigation system that localized the tumor lesion in real time and protected the internal carotid artery from irradiation [117].

A study on the quality of life, overall response rate, overall survival, and adverse events of nine patients with unresectable locally advanced or locally recurrent head and neck carcinoma (LA/LR-HNC), treated with head and neck PIT (HN-PIT) between 20 January 2021 and 30 April 2022, was conducted and published by Okamoto et al. (2022). The results show that no important changes were found in the variables investigated for quality of life, the safety profile was satisfactory, and the positive response rate was 89% [118].

Idogawa et al. (2023) reported their experience with two cases diagnosed with locally rHNSCC successfully treated with PIT. The first case concerned a 63-year-old woman with nasopharyngeal squamous cell carcinoma (NPSCC) that was negative for Epstein-Barr virus-encoded small RNA by in situ hybridization. Under radiotherapy, she had a complete response, but after 6 months, a relapse appeared on the posterior wall of the nasopharynx, histologically confirmed as SCC. She then received Cetuximab sarotalocan sodium I.V. at the standard dose of 640 mg/m^2^ and PIT for 5 min 33 s; 5 weeks after lesion illumination with a 34 mm front diffuser, the tumor completely disappeared. After 6 months the first relapse appeared and then others, so four PIT sessions with devices adapted to the position of the lesion were performed, and in the end, the response was complete and was maintained for 7 months while the patient was monitored. The second case involved a 47-year-old man with a history of NPSCC and metastases in the liver and ileum, for which he received multimodal therapy (surgery, concomitant chemoradiotherapy, and systemic chemo-immunotherapy), under which all lesions disappeared. Even under immunotherapy, a local recurrent lesion of 15 mm extended from the posterior wall to the left, and right lateral ones appeared after 18 months of therapy. Cetuximab sarotalocan sodium was administered, and after surgical interventions, four PIT sessions were administered with difficulty in the affected areas, followed by systemic chemotherapy, and finally good local control was achieved [119].

Good local control can be achieved with PIT, but the therapy is recent, with many unknowns. Additional data from more treated cases are needed [119].

Locally administered PIT for the treatment of superficial lesions with an external light beam has an effective depth of light penetration limited to <10 mm. To stimulate PS in deep tumors or tumors with thicknesses greater than 10 mm, laser fibers are inserted through needles or catheters, where the fibers can be cut flat or terminated with a cylindrical diffuser to provide light also in regions perpendicular to the fiber axis. The end of the cylindrical diffuser varies between 0.5 and 7 cm. Cylindrical diffuser fibers are equipped with a tip that emits light laterally, from one to several centimeters. Cylindrical diffusers emit light at their distal end radially from the fiber core, and this electromagnetic radiation is perpendicular to the fiber axis. Frontal diffusers are used for illumination of superficial tumors to a depth of 1 cm below the skin or mucosal surface, and cylindrical diffusers arranged in needles or catheters are used for deep interstitial approaches > 1 cm from the skin or mucosal surface of tumors [76,120,121,122,123,124].

#### 3.3.3. PIT in Unresectable Recurrent Maxillary Sinus Cancer

Currently, tumors surrounded by bones, such as those in cavities, are more difficult to treat, and the results are modest. Koyama et al. (2023) present the results of PIT in a 56-year-old man diagnosed with unresectable recurrent maxillary sinus cancer, initially undergoing radiotherapy and chemotherapy, who relapsed 6 months after the end of treatment. Chemotherapy was resumed for approximately another year, but the tumor progressed and destroyed the bone structure of the anterior wall of the maxillary sinus. To maximize the safety and efficacy of the therapy, which lasted 1 h and 52 min, two PIT-guided navigation systems were used, one neurosurgical and the second, CT-guided. This ingenious method reduced tumor volume and necrosis without adverse events [125].

#### 3.3.4. PIT in Locally Recurrent Nasopharyngeal Squamous Cell Carcinoma, Positive for Epstein-Barr Virus

PIT for advanced or locoregionally recurrent unresectable head and neck cancer (HNC-PIT) is a recently applied local method that targets EGFR on the surface of cancer cells, and although PIT for NPSCC has been practiced in Japan since September 2020, the results remain unclear. Omura et al. (2023) reported the evolution of a 77-year-old patient, diagnosed with locally recurrent NPSCC, positive for Epstein-Barr virus-encoded small RNA by in situ hybridization, which was treated with concurrent chemoradiotherapy. After 14 months, a local recurrence occurred. This time, the patient received HNC-PIT assisted by transnasal endoscopy for the local recurrence. Seven months after treatment with HNC-PIT, the patient was doing well, without relapses or adverse events. Currently, local recurrence of NPSCC creates great difficulties for minimally invasive surgical approaches, but rescue may come through HNC-PIT [126].

#### 3.3.5. Post-Illumination Pain in Photoimmunotherapy Applied to Head and Neck Cancers

Because pain is the most unpleasant and common adverse reaction to PIT administered to patients with HNC, Shibutani et al. (2023) conducted a retrospective study of five patients who received PIT at the National Cancer Center Hospital East between January 2021 and June 2022 using medical records. The results showed that all patients reported intense pain after PIT, regardless of the illumination method. Pain was assessed on a numerical scale, and from the collected data, it was estimated that the highest level was on the first day immediately after, or at 1 h post-PIT illumination, with mean scores between 6.8 and 7.8 for the frontal and cylindrical illumination techniques, respectively; the pain decreased rapidly on the second day. Given these data, the authors suggest that a pre-established protocol for pain management is needed. This research has some limitations because the study is retrospective and based on a very small number of cases [127].

#### 3.3.6. Eligibility for Photoimmunotherapy in Head and Neck Cancers

Shinozaki et al. (2023) conducted a retrospective study reviewing the medical records of 246 patients at the National Cancer Center Hospital East from January 2016 through December 2020 who started systemic therapy for advanced or recurrent HNC. The authors investigated the essential qualities of patients with HNC to determine whether they were eligible, potentially eligible, or ineligible for treatment with PIT. Only nine patients were considered eligible, but of the nine patients considered eligible under first-line systemic therapy, four no longer met the conditions for PIT because the disease had progressed. The study concludes that for locally advanced or recurrent unresectable HNC, PIT should be considered before, during, and not just after systemic therapy, because it can greatly change the patient’s condition [128].

#### 3.3.7. Immunogenic Cell Death and Changes in Serum DAMPs and Cytokines/Chemokines During NIR-PIT

After NIR-PIT administration, ICD occurs within the first minutes with the release of cytoplasmic material, including damage-associated molecular patterns (DAMPs) and other signaling molecules that will induce the activation of local immune cells [113,129].

Between November 2021 and October 2022, Ishihara et al. (2024) from Aichi Cancer Center Hospital in Nagoya, Japan, studied serum samples collected 1 day before NIR-PIT and 1–3 days after the treatment to track changes in DAMPs (calreticulin, Hsp70, ATP, HMGB1, etc.), whose appearance on the surface of dying cells or in the TME helps determine whether cell death is immunogenic. DAMPs are already known to stimulate DC maturation and trigger cytokine release. The authors also determined the levels of cytokines and chemokines and their relationship to treatment outcomes as well as possible adverse reactions in five HNSCC patients who received a total of seven sessions. The results show that serum HMGB1 levels increased after NIR-PIT in all cases except for one patient who did not respond clinically to therapy. The chemokines macrophage inflammatory protein 1 alpha (MIP-1α/CCL3) and macrophage inflammatory protein 1 beta (MIP-1β/CCL4) reached high levels 1–3 days after therapy. Significant increases in CCL3 and CCL4 after NIR-PIT showed that DAMPs including HMGB1 and Hsp70 have the ability to stimulate immune cells in the TME. A low pre-treatment neutrophil-to-lymphocyte ratio (NLR) was associated with a better response to therapy and survival. Two patients with a high NLR died within 8 months, and another had a poorer response to treatment. Therefore, the NLR ratio could predict response to treatment and survival in patients treated with NIR-PIT. The limitations of the study are that peripheral blood samples were used, and not local tumor tissues, to assess the production of DAMPs and cytokines/chemokines; the adaptive immune response was not investigated; and the number of patients was small [130].

#### 3.3.8. NIR-PIT Combined with ICI Therapy for Unresectable rHNC Could Improve the Anticancer Effects

Hanyu et al. (2024) reported the case of a 75-year-old man with known left mandibular gingival cancer stage 4, squamous cell carcinoma who initially underwent surgical resection, then radiotherapy, and after 1 year and 3 months, presented with a recurrence in the reconstructed area of the left middle pharynx, distant from the carotid artery, so he was considered eligible for PIT. After administration of Cetuximab sarotalocan sodium (640 mg/m^2^) I.V., the pharynx was illuminated posteriorly in four points with a 20 mm cylindrical diffuser, and the superficial lesions were irradiated with a 30 mm frontal diffuser. The local lesion recurred after a total of three sessions of PIT and ICI; the residual tumor was not eradicated. In this situation, immunotherapy with a monoclonal antibody from the PD-1/PD-L1 signaling pathway blocking class of drugs (Pembrolizumab) was initiated. Recurrent lesions disappeared 2 months after the first dose of pembrolizumab and were no longer visible on CT scan more than 12 months after treatment initiation, and the patient continues to receive this drug. In conclusion, the patient had a complete response after PIT followed by ICI immunotherapy [131].

Hirakawa et al. (2024) undertook a study to evaluate the effects of combining NIR-PIT with ICI therapy for unresectable HNSCC. Five patients with unresectable HNSCC who received NIR-PIT between January 2022 and April 2024 were enrolled in the study. Patients with recurrent unresectable HNSCC with a history of prior radiotherapy with distant metastases, those with loco-regional metastases in the bone structures at the base of the skull, mandible, maxilla, or prevertebral muscle, as well as those with tumors located near the carotid artery (within 10 mm of the carotid artery) who had a high risk of fatal bleeding after treatment were excluded from the study. Patients received I.V. Cetuximab sarotarocan sodium (640 mg/m^2^) for 2 h, then after 20–28 h of protection from sunlight in a room with illumination below 120 Lux (lx), NIR-PIT illumination was administered. Tumors were irradiated with diffusers according to tumor location, shape, and size using the BioBlade^®^ laser system. Of the five patients, four received a combination of NIR-PIT and pembrolizumab, administered IV at a dose of 400 mg every 6 weeks. This study demonstrated the best response rate of 60% in three patients who received ICI therapy. Local pain of grade 1 or 2 was the most common adverse effect, lasting for 1–2 days after surgery, in all patients. Severe adverse reactions (grade 3), namely trismus, pharyngocutaneous fistula, and pneumonia, were present in three cases (42.9%), but did not worsen during ICI therapy. At long-term follow-up (157–845 days), four areas with targeted lesions did not have recurrence, unlike three others that showed relapses. All five patients survived, of which three were free of any disease symptoms, indicating that NIR-PIT and ICI therapy are suitable for unresectable recurrent HNC [132].

The favorable response rate in this study with NIR-PIT and ICIs was consistent with previous studies that reported a rate of 43.3–100%, in contrast to those with ICI therapy alone, where the percentage was still moderate, at 15.7–27.3% [100,114,118,133,134].

The high favorable response in NIR-PIT is due on the one hand to the destruction of cancer cells directly with laser, followed by the activation of tumor-specific immunity [135].

Distant (abscopal) anticancer activity, observed in immunocompetent mouse models by shrinking distal, unilluminated tumors, proves the value of PIT in stimulating local and peripheral T-cell responses. Therefore, PIT should be associated with other immunotherapies to obtain an enhanced and persistent anticancer effect [136].

Koyama et al. (2024) presented the case of a 56-year-old man with squamous cell carcinoma of the maxillary sinus who received radiotherapy and chemotherapy in three cycles of Cisplatin at a total dose of 300 mg/m^2^, and approximately 6 months after the end of treatment, CT documented tumor recurrence involving the mid-cranial base. In this case, the ICI drug (anti-PD-1 antibody), Pembrolizumab, was used at a dose of 200 mg/kg body weight every 3 weeks as first-line treatment. But the disease could not be controlled 5 months after therapy (no tumor shrinkage, progressive disease). Then, a sequential three-drug combination was administered as second-line chemotherapy with Cetuximab, Paclitaxel, and Carboplatin. After four sessions of NIR-PIT, a reduction in tumor volume was noted; then, the tumor began to grow rapidly again in the 2 months following the last PIT session, and consequently, Pembrolizumab was re-administered. Twelve months after the final Pembrolizumab administration, Nivolumab I.V. was introduced at a dose of 240 mg/kg body weight every 2 weeks, for 30 min. Tumor volume reduction was achieved immediately after chemotherapy was resumed, and 5 months later, the tumor disappeared. The positive effects were maintained 8 months after the start of Nivolumab chemotherapy, without severe adverse effects. If the patient was initially refractory to ICI, he became responsive following re-administration of ICI after the NIR-PIT sessions. The authors believe that NIR-PIT would activate the host’s anticancer immunity, increase the efficiency of ICI therapy, and overcome the resistance of tumor cells to anti-PD-1 [137].

#### 3.3.9. Emergency Tracheostomy After Head and Neck Photoimmunotherapy

Since the approval of HN-PIT in Japan for unresectable, locally advanced, and locally recurrent HNC, the number of treatments has increased to over 350, and the number of clinical trials and case reports has been continuously growing. One of the specific problems with this therapeutic modality is the occurrence of laryngeal edema, which can be life-threatening, and therefore, the patient must be informed and sign the consent for prophylactic or emergency tracheostomy. Preventive tracheostomy is recommended in all patients with tumors located in the laryngeal area (laryngeal, hypopharyngeal lesions and cervical lymph node involvement adjacent to the larynx). However, the frequency of laryngeal edema is incomprehensible when addressing areas distant from the larynx, such as the paranasal sinuses, nasopharynx, oral cavity, and oropharynx [109,116,128].

Okamoto et al. (2024) have reported their experience with 23 patients who received 44 cycles of HN-PIT between January 2021 and October 2023. Of the 23 patients, two who were not considered at risk and did not undergo preventive tracheostomy presented with severe symptoms of laryngeal edema, requiring emergency tracheostomy. The first patient, a 70-year-old man, presented after surgery and radiotherapy for stage III HNSCC (mandibular gingival cancer in the oropharynx), a local recurrence in the lateral-to-posterior wall of the left oropharynx. HN-PIT illumination in cycle 1 was performed through four cylindrical diffusers with a diameter of 20 mm and a frontal diffuser with a spot diameter of 30 mm. Laryngeal edema occurred during therapy and was treated with injectable steroid preparations; the edema disappeared within 4 days. In the second cycle, the remaining area was illuminated four times with a frontal diffuser having a spot diameter of 30 mm [138].

The second patient was also a 70-year-old man with HNSCC (p16 negative, stage III), at the level of the upper wall of the oropharynx. The patient had a history of resected tongue cancer, mediastinal lymph node metastases, laryngeal, hypopharyngeal, and esophageal cancer followed by surgical resections, chemoradiotherapy or radiotherapy, and various reconstructions. He presented with a newly diagnosed oropharyngeal cancer at the level of the palate and buccal mucosa, after unsuccessful radiotherapy. HN-PIT was administered three times using a frontal diffuser with a spot diameter of 38 mm. The patient developed respiratory failure induced by severe laryngeal edema 6 h post-therapy, with swelling of the cheek, lip, and right oral cavity, which is why an emergency tracheostomy was performed. The mechanism by which laryngeal edema occurred during HN-PIT is not yet fully understood, especially since the illumination was performed at a distance from the larynx. The HN-PIT method still has many unknowns, which will be elucidated over time through clinical practice [138].

Oropharyngeal cancer caused by papillomavirus infection would have a better evolution under chemoradiotherapy, but relapses occur in 30–40% of cases in stage III/IV, even after primary chemoradiotherapy, and ultimately has a reserved prognosis. Since the base of the tongue participates in the activity of phonation and swallowing, the surgical method is avoided, as there is a risk of complications that endanger the patient’s life, and at the same time, the success rate is only 46% in treated cases. Radiotherapy and classical chemotherapy have not given satisfactory results, and molecularly targeted drugs and ICIs used for recurrent cases are effective only in reducing the number of cancer cells [139,140].

Tamagawa et al. (2024) reported the case of a 60-year-old patient with an endoscopically detected lesion at the base of the tongue (histological examination: squamous cell carcinoma) that recurred 12 years after concurrent chemoradiotherapy for oropharyngeal cancer. The patient’s personal pathological history includes partial glossectomy on the right side for lingual cancer and transoral resection of left buccal mucosa cancer 5 years ago. NIR-PIT was chosen because the patient was at high risk of complications due to severe dysphagia and open wounds after surgical interventions. Twenty-four hours after administration of Cetuximab sarotalocan sodium at the standard dose of 640 mg/m^2^, NIR-PIT illumination was performed with a PDT semiconductor laser and four frontal diffuser probes, for 5 min. Following NIR-PIT illumination, a severe edema of the epiglottis mucosa occurred, for which a cortisone preparation was administered, and a tracheostomy was performed prophylactically to avoid respiratory failure. After therapy, at 2 and 10 months, respectively, the tumor did not recur or metastasize. This case illustrates the therapeutic efficacy and appropriate management of pain and respiratory failure [141].

Nasopharyngeal carcinoma is a form of cancer that poses great therapeutic problems precisely because of its anatomical position, and therefore, the preferred options are radiotherapy or chemoradiotherapy. Due to advances in recent decades, the surgical approach in patients with nasopharyngeal carcinoma in the early and middle stages, through complete resection, has gained priority over radiotherapy [142,143,144].

As an innovative treatment method, NIR-PIT has become the technique of choice in cases of nasopharyngeal carcinoma that do not respond to chemoradiotherapy, due to its nature of being surgically unresectable and the lack of standard treatment. Until the invention of PIT as an alternative therapy, all patients with failed conventional treatment had no other option than palliative care. Kushihashi et al. (2024) reported the results of PIT treatment in a patient with radiation-induced nasopharyngeal carcinoma, after an ineffective combination of chemoradiotherapy. This was a 34-year-old patient with a history of parapharyngeal rhabdomyosarcoma, for which she received radiotherapy at the age of 10, and who, at the time of PIT administration, had a diagnosis of cancer localized to the nasopharynx, without distant metastases (SCC, cT1N0M0, stage I). After administration of Cetuximab sarotarocan sodium (640 mg/m^2^), the lesion was illuminated with a 20 mm frontal diffuser. On the first postoperative day, respiratory dysfunction phenomena with reduced blood oxygen saturation and difficulties with oral intubation occurred, which is why an emergency tracheostomy was performed at the patient’s bedside. The subsequent evolution was good, and the patient was discharged on postoperative day 12. Among the inconveniences that occurred during PIT therapy, we mention bleeding after tumor necrosis and emergencies induced by airway damage due to laryngeal edema. Prophylactic tracheostomy has become a practice when PIT is administered for tumors located at the root of the tongue, hypopharynx, and larynx. In the presented case, the authors mention the possibility of complications related to acute respiratory failure during PIT administration, regardless of the site of illumination, as well as the importance of careful monitoring and immediate therapy [145].

Although the exact mechanism underlying the generation of post-PIT mucosal edema remains unknown, the authors discussed the following picture of related phenomena: after PIT induces tumor cell necrosis, DAMPs are released, which stimulate cytokine production and trigger an inflammatory response, which will lead to the appearance of laryngeal edema as a result of the subsequent increase in vascular permeability. It is possible that excessive illumination of the area adjacent to the soft palate induces strong vasodilation with extensive fluid accumulation and edematous complications of the anatomical structures of the upper airways [138,146,147,148].

#### 3.3.10. Preoperative Simulation and NIR-PIT Illumination with HMD-MR Technology

Concurrently with the rapid evolution of technology that combines virtual reality (VR) and augmented reality (AR), the medical field has been involved in the implementation of this innovative mixed reality (MR) technology, especially in defining precise medical diagnoses, as well as in surgical interventions. AR reproduces the phenomenon by which it is feasible to superimpose a digital (virtual) reality over the real, concrete universe, seen with the naked eye. The first head-mounted VR/AR display system connected to a computer, then called the Sword of Damocles, was invented in 1968 by Ivan Sutherland and his student Bob Sproull. The term “augmented reality” was defined by the distinguished physicist Thomas Caudell, during his tenure with the Boeing Company in the 1990s, who, through his research and innovative applications of AR technology, provided an extraordinary solution to the problems generated by human–computer interaction, thus helping to pave the way for the invention of digital operating systems in industry, business, medicine, and individuals alike. Today, AR operating systems seamlessly reduce the boundaries between reality and digital content and provide unparalleled platforms for immersive training and simulations, revolutionizing industry, manufacturing, commerce, aviation, software, art, tourism, healthcare, etc. Now, medicine is one of the many beneficiaries of MR technology, especially in surgery by facilitating surgeon training, simulation, and navigation. Through these applications, the surgeon has the opportunity to have visual control over anatomical structures and the exact position of tumors in three-dimensional space as if they were visible through the skin. Using intraoperative MR techniques, the surgeon can perform procedures by using a head-mounted display (HMD) that visualizes 3D holograms (computer graphic models) of the patient [149,150,151,152,153,154,155].

Okada et al. (2024) reported the case of an 86-year-old man with recurrent oropharyngeal cancer who had a history of laryngeal cancer with total laryngectomy and radiotherapy. The patient had a tumor located in the posterior wall of the oropharynx and an enlarged right cervical lymph node. Since the patient had a compromised general condition, he was selected for NIR-PIT. Following CT and 18-Fluoro-deoxyglucose positron emission tomography (FDG-PET) or FDG-PET/CT imaging, a personalized 3D model of the head and neck was generated for the patient, and the hologram was loaded on the HMD; the images were then processed on Digital Imaging and Communications in Medicine (DICOM) in a 3D Slicer, and the anatomical structures were converted into stereolithography (STL) files, which were then uploaded to Holoeyes XR (Holoeyes Inc., Minato City, Tokyo, Japan), an online service for online STL file viewing. Operators used Microsoft HoloLens2 (Microsoft Corporation, Redmond, WA, USA) to view the 3D virtual images as holograms and study both the real image and the virtual images loaded as holographic media, which could be moved, rotated, and zoomed in by gestures. After transfer, distinct anatomical parts were assigned specific colors or became translucent with different levels of translucency to explore the internal anatomical shapes in depth. Cetuximab sarotalocan I.V. was used, and the tumor was illuminated the following day under general anesthesia, through cylindrical diffusers inserted into needle catheters. The patient presented with local grade 1 pain and mild pharyngeal edema that was resolved within a week. A second illumination session was repeated 2 months later, and no recurrence was observed on endoscopy and MRI, 3 months after the last NIR-PIT session. Preoperative simulation and illumination were guided for the first time during surgery by HMD-MR technology, and thus, the NIR-PIT efficacy was improved. HMD-MR technology allowed the integration of 3D holograms into the patient’s real surgical environment and was extremely useful in the ideal placement of catheters by providing visual support for real anatomical structures, along with endoscopic snapshots during the intervention [156].

#### 3.3.11. Lemierre’s Syndrome, a Rare Complication of NIR-PIT

NIR-PIT is considered the treatment of choice for patients with unresectable, locally advanced, or locally recurrent HNC in whom salvage surgery is difficult. Promising clinical data have already been accumulated since the implementation of this innovative method, and there is hope that the technique will be refined and advanced [126,157].

Common complications after this therapeutic intervention are pain and local pharyngo-laryngeal edema; a rare adverse reaction, Lemierre’s syndrome (LS), has recently been reported. LS is known as a rare disease that occurs after a bacterial infection of the pharynx or tonsils, which sometimes progresses to the lateral pharyngeal areas of the neck, with the development of septic thrombophlebitis of the internal jugular vein(s). The infection may be associated with anaerobic organisms and may cause life-threatening septicemia. Because oropharyngeal infections are common and benign, diagnosis is often delayed [158,159,160,161].

Nishimura et al. (2024) treated a 68-year-old patient with nasopharyngeal cancer (right lateral wall of the nasopharynx SCC, stage III) who had been treated with chemoradiotherapy, the outcome of which was considered unsatisfactory, which is why he received HN-PIT after cervical lymph node dissection. After HN-PIT therapy, nasopharyngeal mucositis appeared and persisted, and over time, the primary tumor shrank. In contrast, 2 months after illumination with HN-PIT, the patient complained of headache, neck pain, fever, tachycardia, and anorexia, for which he was urgently hospitalized. Biological data confirmed systemic inflammatory response syndrome and positive blood cultures for *Fusobacterium necrophorum* and *Prevotella denticola*, and contrast-enhanced CT revealed a bilateral disseminated venous thrombus extending to the vertebral veins, bilateral brachiocephalic veins, right internal jugular vein, and pterygoid venous plexus. Based on signs of mucositis, thrombophlebitis on CT, and inflammatory syndrome after NH-PIT illumination, Lemierre’s syndrome was diagnosed. After therapy with broad-spectrum antibiotics and those directed against the detected Gram-negative anaerobic organisms to which they were sensitive, the symptoms were resolved. HN-PIT is a highly destructive therapy that can cause mucositis, which in turn can promote the development of LS, a pathological event that endangers the patient’s life if it occurs and requires increased attention from the medical team. The care of mucositis after HN-PIT is essential for maintaining the barrier function of the mucosa [162].

The explanation for this complication would be the illumination of the fragile mucosa with HN-PIT at the level of the recurrent lesion and unhealed wounds after surgery, which would have provided an entry gate for infectious agents, while prolonged postoperative mucositis would have favored the occurrence of LS. Data from the specialized literature draw attention to the increased risk of LS after HN-PIT illumination of tumors in the nasopharynx, oropharynx, and oral cavity. At the same time, it is possible that some patients who develop septic infectious complications after HN-PIT should be suspected of LS. All surgeons involved in the administration of HN-PIT should be advised and trained to recognize LS, so as to apply an appropriate early treatment, since the mortality rate in this pathology is high [116,162,163,164].

**Table 3:** Photoimmunotherapy in patients with unresectable, locally advanced or locally recurrent head and neck cancer.

References	Study Characteristics	Type of Cancer	No. Patients	Drugs	PIT Laser Protocol	Efficacity	Adverse Events (AEs)
Ref. [109]	Single-center, open-label, phase I study.	rHNSCC	3	RM-1929 640 mg/m^2^ (cetuximab sarotalocan) Cetuximab-IR700DX conjugate; Light-activatable dye (IRDye 700DX).	50 J/cm^2^ superficial illumination. 100 J/cm fiber diffuser length for interstitial illumination. Approximately 5 min for each treated region. Class IV laser precautions were required for delivery of illumination.	Significant clinical response and good safety.	AEs were mild to moderate and transient grade 3 for application site pain.
Ref. [110]	Case report	Locally recurrent nasopharyngeal carcinoma	1	Cetuximab sarotalocan sodium (640 mg/m^2^) + PIT	Cylindrical diffuser and frontal diffusers. Light irradiation using a laser (form Rakuten Medical, Tokyo, Japan).	Complete response to PIT	None.
Ref. [114]	Review and two case reports	HNC Case 1: Left oropharyngeal tumor. Case 2: Cancer of the base of tongue.	2	Day 1: Intravenous administration of RM-1929 at a dose of 640 mg/m^2^ by intravenous infusion over 2 h. + NIR-PIT	Day 2: Tracheostomy before laser illumination. Laser illumination was performed with BioBlade^®^ laser system (Rakuten Medical, Inc., San Diego, CA, USA). For deep lesions or thick lesions, 20 mm length cylindrical diffusers were used, and for superficial lesions, a frontal diffuser was used.	Good results without functional complications.	without serious side effects.
Ref. [116]	Case report	Recurrent laryngeal squamous cell carcinoma	1	Cetuximab sarotalocan sodium (640 mg/m^2^) + PIT	Laser irradiation using cylindrical diffusers and a PDT semiconductor laser (Rakuten Medical). Irradiation time: First session, 8 min and 20 s. Second session (6 weeks after the first), 9 min and 43 s.	PIT response was considered partial after the first session.	Grade 2 acne-like skin rash.
Ref. [117]	Case report	Maxillary gingival carcinoma with a recurrent lesion deep in the lateral pterygoid muscle.	1	Cetuximab sarotalocan sodium (640 mg/m^2^) + PIT with 690 nm red light illumination.	PDT with semiconductor laser (BioBrade^®^ laser) and PDT with semiconductor laser probes (BioBrade^®^ cylindrical diffuser, BioBrade^®^ frontal diffuser, and BioBrade^®^ needle catheter; Rakuten Medical, Tokyo, Japan). Intra-tissue irradiation: cylindrical laser beam with a radius of 10 mm Irradiation time was 9 min and 43 s.	Complete response.	None.
Ref. [118]	Evaluation of the quality of life of 9 patients with LA-HNC or LR-HNC	Unresectable locally advanced or locally recurrent head and neck carcinoma	9	Cetuximab sarotalocan sodium (640 mg/m^2^) + HN-PIT	Optimal laser light intensity was 50 J/cm^2^ for superficial lesions, and 100 J/cm for deep lesions.	HN-PIT may prolong overall survival. The safety profile was satisfactory, with a positive response rate of 89%.	Mucositis (89%); Edema of the larynx (33%); Hemorrhage (22%); Acneiform rash (11%).
Ref. [119]	Two case reports	Recurrent NPSCC	2	Cetuximab sarotalocan sodium (640 mg/m^2^) + PIT	Case 1: A frontal diffuser (34 mm from the posterior wall) illuminated the whole tumor, for 5 min 33 s. Case 2: Two 100 mm needle catheters were used to perforate the posterior wall, through each nasal cavity, a total of four. A 20 mm cylindrical diffuser was inserted into each catheter and illuminated. A residual tumor was present, and a second PIT was planned.	Case 1: Five weeks postoperatively, the tumor had disappeared completely. Case 2: Patient underwent four PIT sessions, followed by systemic chemotherapy, and survived with cancer 1 year after the first PIT.	Grade 2 adverse reactions: pain, laryngeal edema, and suspected osteomyelitis.
Ref. [125]	Case report	Maxillary sinus cancer (rHNC)	1	Cetuximab sarotalocan sodium (640 mg/m^2^) + CT-guided PIT with surgical navigation	1 h and 52 min	Tumor necrosis and volume decreased. CT guidance has helped maximize the safety and efficacy of PIT for rHNC.	Pain
Ref. [126]	Case report	Locally recurrent NPSCC	1	Cetuximab sarotalocan sodium (640 mg/m^2^) + HNC-PIT assisted by transnasal endoscopy	Laser illumination was for 5 min and 33 s.	Patient without recurrence or adverse events	Hypoxia during the intravenous administration of Cetuximab.
[127]	Retrospective study. A Case Series.	HNSCC	5	Cetuximab sarotalocan sodium (640 mg/m^2^) + PIT	5 min for each treated area.	Pain: first day immediately after, or at one-hour post-PIT illumination (mean scores 6.8–7.8 for the frontal and cylindrical illumination).	Acute and transitory pain. A plan for postoperative pain management is needed in PIT.
[128]	Retrospective study	Unresectable advanced or recurrent HNC	246	Cetuximab sarotalocan sodium and the BioBlade laser system. The tumor was illuminated with laser light (690 nm) on day 2, resulting in tumor necrosis. Cylindrical diffusers in needle catheters for subcutaneous (submucosal) lesions and a front diffuser for superficial lesions.	HN-PIT evoked before, during, and after systemic therapy.	In patients with relapsed metastatic HNSCC, HN-PIT is a promising alternative because incurable locoregional disease remains a strongly adverse prognostic factor. HN-PIT should be considered as a treatment option before systemic chemotherapy, so as not to miss the right moment for treatment.	Some immune-related adverse events induced by systemic therapy. 50% of eligible patients became ineligible for HN-PIT due to disease progression after systemic therapy.
Ref. [130]	Brief communication	HNSCC	5	Cetuximab sarotalocan sodium (640 mg/m^2^) + NIR-PIT	Seven sessions of NIR-PIT.	DAMPs and serum cytokine/chemokine levels were increased after NIR-PIT. Baseline NLR may predict patient outcomes in response to NIR-PIT.	Grade 4 laryngeal edema; fistula. Pain and edema in all patients.
Ref. [131]	Case report	Local recurrent mandibular gingival cancer (HNC)	1	Cetuximab sarotalocan sodium (640 mg/m^2^) + PIT + ICI therapy with Pembrolizumab.	Cycle 1: 4 spots with a 20 mm cylindrical diffuser, and a frontal diffuser (30 mm diameter). Cycle 2: Residual superficial lesions were irradiated at 4 locations with the same frontal diffuser. Cycle 3: Residual lesions were irradiated at 6 locations using 40 mm and 20 mm cylindrical diffusers, and the superficial lesions at 2 locations using frontal diffusers (38 mm spot diameters).	ICI could be successfully administered in cases with advanced disease, after HN-PIT. The patient remained in clinical remission 1 year after therapy.	ICI can be effective after HN-PIT with no AEs.
Ref. [132]	Feasibility study	Unresectable rHNSCC	5	Cetuximab sarotalocan sodium (640 mg/m^2^) followed by NIR-PIT + ICI therapy with Pembrolizumab.	Tumors over 10 mm thick were illuminated with a cylindrical diffuser (laser light over a 15 mm radius), and superficial ones up to 10 mm with a frontal diffuser. Best observed response rate was 100%. Of the total of 7 targeted lesions: 3 complete responses, and 4 partial responses.	Combining NIR-PIT and ICI therapy is safe and effective as a promising master plan for locally incurable advanced or recurrent HNSCC. NIR-PIT with Pembrolizumab has proven effective and safe.	Grade 1 or 2 pain in all patients for 1–2 days postoperatively. Grade 3 pneumonia, pharyngeal skin fistula, and trismus in 42.9% of cases.
Ref. [137]	Case Report	Local recurrent maxillary sinus cancer	1	NIR-PIT + Pembrolizumab. Second-line chemotherapy with: Cetuximab, Paclitaxel, and Carboplatin, followed by Nivolumab.	4 NIR-PIT sessions	NIR-PIT is a potential stimulator of host anticancer immunity, ICI efficacity, and circumvention of ICI resistance.	Fistula between the maxillary sinus and the skin.
Ref. [138]	Case reports	rHNSCC Case 1: local recurrence of mandibular gingival carcinoma. Case 2: carcinoma of the mid-pharynx.	2 cases with severe laryngeal edema post HN-PIT	Cetuximab sarotalocan sodium (640 mg/m^2^) + HN-PIT	Cylindrical diffuser illuminated the tissue with a 10 mm radius and 100 J/cm; the energy density for frontal diffuser was 50 J/cm^2^, with a distributed depth of <10 mm.	During HN-PIT therapy, patients may go through several stages, one of which is laryngeal edema, for which an emergency tracheostomy was performed.	Two cases of laryngeal edema with emergency tracheostomy.
Ref. [141]	Case report and Review	Locoregionally recurrent oropharyngeal SCC at tongue base.	1	Cetuximab sarotalocan sodium (640 mg/m^2^) + NIR-PIT	Laser illumination for 5 min with four cylindrical diffusers.	Success of NIR-PIT without post-operative dysfunction in a case of locoregionally recurrent oropharyngeal cancer refractory to chemoradiotherapy.	Severe mucosal edema from the epiglottis to the tongue base and grade 3 or higher pain. A prophylactic tracheostomy was performed under general anesthesia.
Ref. [145]	Case report	Nasopharyngeal carcinoma (SCC, cT1N0M0, Stage I)	1	Cetuximab sarotalocan sodium (640 mg/m^2^) + PIT	Transoral laser illumination with a 20 mm frontal diffuser.	Acute respiratory failure can be life-threatening and should be considered as a potential adverse effect of PIT.	Laryngeal edema required emergency tracheostomy.
Ref. [156]	Case report	Recurrent oropharyngeal cancer. Patient with compromised general health.	1	Cetuximab sarotalocan sodium (640 mg/m^2^) + 2 NIR-PIT sessions, using HMD-MR technology.	From CT and FDG-PET/CT images, a customized 3D HN model specific to the patient was generated. Four needle catheters were inserted into the tumor, and this was illuminated through the cylindrical diffusers.	First application of HMD-MR technology, which optimized the NIR-PIT effect.	Grade 1 local pain and mild pharyngeal edema (which disappeared within 7 days).
Ref. [162]	Case report	Nasopharynx SCC, stage III	1	Cetuximab sarotalocan sodium (640 mg/m^2^) + HN-PIT with 690 mm laser light to specifically damage tumor cell membranes + post-antibiotic therapy	Cylindrical diffusers were inserted into the tumor	Tumor has shrunk.	Postoperative mucositis and Lemierre’s syndrome (pathology with high mortality rate).

## 4. Conclusions

Head and neck squamous cell carcinoma is the sixth most common cancer worldwide and accounts for almost 90% of all head and neck cancers.

The current treatment protocol for unresectable recurrent or metastatic HNSCC consists of surgical techniques, radiotherapy, chemotherapy drugs, or chemotherapy associated with EGFR-targeted therapy (cetuximab) and, recently, ICIs.

ICIs used in clinical practice today should sustainably reduce the volume of various types of tumors with much fewer side effects and in a higher percentage of patients, compared to other immunotherapies.

However, even with the use of new biomarkers predictive of response to anti-PD-1/PD-L1 agents, not all patients have favorable outcomes, and the emergence of de novo and acquired resistance is noted in many patients, which is of particular concern.

To enhance the effects of ICIs and to make progress in the elucidation of the mechanisms underlying the development of resistance to the administered agents, it is essential to discover new treatment modalities, or combinations thereof, that are cost-effective and harmless.

NIR-PIT, as a recently developed method of molecularly targeted antitumor phototherapy, induces selective and immediate killing of cancer cells, but at the same time strongly stimulates the body’s own anti-tumor immunity, which increases its effect.

Phthalocyanine dye IRDye 700DX (IR700), which is light-activated at a wavelength of 690 nm, was conjugated with Cetuximab, thus obtaining the experimental drug ASP-1929, which became a key component for PIT in selectively targeting the membrane of tumor cells that overexpress EGFR, and killing these cells.

NIR-PIT applied in the therapy of unresectable, locally advanced or locally recurrent head and neck cancer, considered palliative, has proven its effectiveness.

NIR-PIT, successfully administered to date in unresectable rHNSCC, brings great hope to patients with these forms of cancer, but studies are still few, the number of patients is small, and the few cases monitored over the long term limit the attestation of the efficacy and safety of this approach.

For future applications, the discovery of biomarkers for diagnosis and treatment optimization, dose prediction, prognosis, and risk stratification for PIT could revolutionize integrative personalized oncology.

The use of artificial intelligence, medical image analysis, and biomedical signal processing through advanced virtual reality, augmented reality, and the implementation of the innovative mixed reality technology, especially in defining precise medical diagnosis, as well as in surgical interventions within photoimmunotherapy, should be successfully and widely implemented.

Harnessing PIT to strengthen its clinical outcomes could represent a significant advance in shaping cancer research to overcome its current limitations.

NIR-PIT is emerging as a highly innovative therapeutic modality for the treatment of unresectable head and neck cancers. It offers a novel mechanism of action and represents a promising option for patients with limited or no viable alternatives. Given its targeted approach and localized cytotoxic effect, NIR-PIT has the potential to significantly improve clinical outcomes and survival rates in this challenging patient population.

For regulatory approval, particularly by the U.S. Food and Drug Administration (FDA), Phase III clinical trials must demonstrate that a new therapy is not only safe and efficacious but also provides a favorable risk–benefit profile in comparison to current standard-of-care treatments. NIR-PIT is currently under investigation with the aim of meeting these stringent requirements.

Moreover, the therapeutic potential of NIR-PIT may be further enhanced through synergistic integration with other transformative advancements in oncology. These include artificial intelligence-driven diagnostics, liquid biopsies for early detection, molecular profiling of tumor DNA, precision medicine strategies, CAR-T cell therapies, and personalized cancer vaccines.

Despite these rapid advances, the biological complexity and adaptability of cancer continue to present formidable challenges. A multidisciplinary, systems-level approach will be essential to fully realize the potential of emerging therapies like NIR-PIT in the broader context of cancer treatment.

## Data Availability

The literature articles used in this review are available from the first author (L.M.A).

## Abbreviations

The following abbreviations are used in this manuscript:

ACT	adoptive cell therapies
AEs	adverse events
Ag(s)	antigen(s)
APC	antibody–photoabsorber conjugate
AR	augmented reality
ASCO	American Society of Clinical Oncology
ASP-1929 (RM-1929)	cetuximab sarotalocan or cetuximab-IRDye 700DX conjugate or Akalux
ATP	adenosine triphosphate
CAR-T cell	chimeric antigen receptor transduced T cell
COVID-19	Coronavirus disease 2019
CRT	calreticulin
CSCC	cutaneous squamous cell carcinoma
CT	computed tomography
CTLA-4	cytotoxic T-lymphocyte associated antigen 4
DAMPs	damage-associated molecular patterns
DC	dendritic cell
DCs	dendritic cells
DDSs	drug delivery systems
DICOM	Digital Imaging and Communications in Medicine
EGFR	epidermal growth factor receptor
EPR	enhanced permeability and retention
ESCC	esophageal squamous cell carcinoma
FDA	U.S. Food and Drug Administration
FDG-PET	18-Fluoro-deoxyglucose positron emission tomography
FDG-PET/CT	CT and 18-Fluoro-deoxyglucose positron emission tomography
HMD	head-mounted display
HMGB1	high-mobility group box 1 protein
HNC	head and neck cancer
HNCs	head and neck cancers
HN-PIT	head and neck photoimmunotherapy
HNC-PIT	photoimmunotherapy for head and neck cancer
HNSCC	head and neck squamous cell carcinoma
HPV	*Human Papilloma Virus*
Hsp70/Hsp90	heat shock proteins 70 and 90
ICB	immune checkpoint blockade
ICD	immunogenic cell death
ICI	immune checkpoint inhibitor
ICIs	immune checkpoint inhibitors
irAEs	immune-related adverse events
IR700	phthalocyanine dye IRDye 700DX
I.V.	intravenous
laCSCC	locally advanced CSCC
LAG-3	lymphocyte activation gene-3
LA/LR-HNC	locally advanced or locally recurrent head and neck carcinoma
LS	Lemierre’s syndrome
lx	lux (luminous flux)
mAb	monoclonal antibody
mCSCC	metastatic cutaneous squamous cell carcinoma
MDSCs	myeloid-derived suppressor cells
mHNSCC	metastatic head and neck squamous cell carcinoma
MIP-1α/CCL3	macrophage inflammatory protein 1 alpha
MIP-1β/CCL4	macrophage inflammatory protein 1 beta
MR	mixed reality
MRI	magnetic resonance imaging
NCBI	National Center for Biotechnology Information
NIR	near-infrared
NIR-PIT	near-infrared photoimmunotherapy
NLM	National Library of Medicine
NLR	neutrophil-to-lymphocyte ratio
NPSCC	nasopharyngeal squamous cell carcinoma
^1^O_2_	singlet oxygen
PD-1	programmed cell death protein 1
PD-L1	programmed cell death ligand 1
PD-L2	programmed cell death ligand 2
PDT	photodynamic therapy
PET	positron emission tomography
PIT	photoimmunotherapy
PSs	photosensitizers
PTT	photothermal therapy
rHNC	recurrent head and neck cancer
rHNSCC	recurrent head and neck squamous cell carcinoma
rLA	recurrent locally advanced
r/m	recurrent/metastatic
ROS	reactive oxygen species
SCC	squamous cell carcinoma
STL	stereolithography
SUPR	super-enhanced permeability and retention
TAMs	tumor-associated M2 macrophages
TCA	tricarboxylic acid cycle
TIME	tumor immune microenvironment
TME	tumor microenvironment
Tregs	regulatory T lymphocytes
US	United States
VR	virtual reality

## References

[1] Siegel R.L., Miller K.D., Wagle N.S., Jemal A. (2023). Cancer statistics, 2023. Cancer Stat..

[2] Cronin K.A., Scott S., Firth A.U., Sung H., Henley S.J., Sherman R.L., Siegel R.L., Anderson R.N., Kohler B.A., Benard V.B. (2022). Annual report to the nation on the status of cancer, part 1: National cancer statistics. Cancer.

[3] Huhulea E.N., Huang L., Eng S., Sumawi B., Huang A., Aifuwa E., Hirani R., Tiwari R.K., Etienne M. (2025). Artificial Intelligence Advancements in Oncology: A Review of Current Trends and Future Directions. Biomedicines.

[4] Ghoreyshi N., Heidari R., Farhadi A., Chamanara M., Farahani N., Vahidi M., Behroozi J. (2025). Next-generation sequencing in cancer diagnosis and treatment: Clinical applications and future directions. Discov. Oncol..

[5] Sathyanarayanan V., Neelapu S.S. (2015). Cancer immunotherapy: Strategies for personalization and combinatorial approaches. Mol. Oncol..

[6] Kato D., Yaguchi T., Iwata T., Morii K., Nakagawa T., Nishimura R., Kawakami Y. (2017). Prospects for personalized combination immunotherapy for solid tumors based on adoptive cell therapies and immune checkpoint blockade therapies. Nihon Rinsho Meneki Gakkai Kaishi.

[7] Martinez M., Moon E.K. (2019). CAR T Cells for Solid Tumors: New Strategies for Finding, Infiltrating, and Surviving in the Tumor Microenvironment. Front. Immunol..

[8] Sharma P., Siddiqui B.A., Anandhan S., Yadav S.S., Subudhi S.K., Gao J., Goswami S., Allison J.P. (2021). The Next Decade of Immune Checkpoint Therapy. Cancer Discov..

[9] Yap T.A., Parkes E.E., Peng W., Moyers J.T., Curran M.A., Tawbi H.A. (2021). Development of Immunotherapy Combination Strategies in Cancer. Cancer Discov..

[10] El-Kadiry A.E., Rafei M., Shammaa R. (2021). Cell Therapy: Types, Regulation, and Clinical Benefits. Front. Med..

[11] Sharma P., Goswami S., Raychaudhuri D., Siddiqui B.A., Singh P., Nagarajan A., Liu J., Subudhi S.K., Poon C., Gant K.L. (2023). Immune checkpoint therapy-current perspectives and future directions. Cell.

[12] Mishra A., Maiti R., Mohan P., Gupta P. (2024). Antigen loss following CAR-T cell therapy: Mechanisms, implications, and potential solutions. Eur. J. Haematol..

[13] Correia J.H., Rodrigues J.A., Pimenta S., Dong T., Yang Z. (2021). Photodynamic Therapy Review: Principles, Photosensitizers, Applications, and Future Directions. Pharmaceutics.

[14] Aebisher D., Czech S., Dynarowicz K., Misiołek M., Komosińska-Vassev K., Kawczyk-Krupka A., Bartusik-Aebisher D. (2024). Photodynamic Therapy: Past, Current, and Future. Int. J. Mol. Sci..

[15] Zhao W., Wang L., Zhang M., Liu Z., Wu C., Pan X., Huang Z., Lu C., Quan G. (2024). Photodynamic therapy for cancer: Mechanisms, photosensitizers, nanocarriers, and clinical studies. MedComm.

[16] Ailioaie L.M., Ailioaie C., Litscher G. (2025). Fighting Cancer with Photodynamic Therapy and Nanotechnologies: Current Challenges and Future Directions. Int. J. Mol. Sci..

[17] Mohiuddin T.M., Zhang C., Sheng W., Al-Rawe M., Zeppernick F., Meinhold-Heerlein I., Hussain A.F. (2023). Near Infrared Photoimmunotherapy: A Review of Recent Progress and Their Target Molecules for Cancer Therapy. Int. J. Mol. Sci..

[18] Mew D., Wat C.K., Towers G.H., Levy J.G. (1983). Photoimmunotherapy: Treatment of animal tumors with tumor-specific monoclonal antibody-hematoporphyrin conjugates. J. Immunol..

[19] Goff B.A., Bamberg M., Hasan T. (1991). Photoimmunotherapy of human ovarian carcinoma cells ex vivo. Cancer Res..

[20] Duska L.R., Hamblin M.R., Miller J.L., Hasan T. (1999). Combination photoimmunotherapy and cisplatin: Effects on human ovarian cancer ex vivo. J. Natl. Cancer Inst..

[21] Molpus K.L., Hamblin M.R., Rizvi I., Hasan T. (2000). Intraperitoneal photoimmunotherapy of ovarian carcinoma xenografts in nude mice using charged photoimmunoconjugates. Gynecol. Oncol..

[22] Fukushima H., Turkbey B., Pinto P.A., Furusawa A., Choyke P.L., Kobayashi H. (2022). Near-Infrared Photoimmunotherapy (NIR-PIT) in Urologic Cancers. Cancers.

[23] Mitsunaga M., Ogawa M., Kosaka N., Rosenblum L.T., Choyke P.L., Kobayashi H. (2011). Cancer cell-selective in vivo near infrared photoimmunotherapy targeting specific membrane molecules. Nat. Med..

[24] Chen D.S., Mellman I. (2013). Oncology meets immunology: The cancer-immunity cycle. Immunity.

[25] Sato K., Sato N., Xu B., Nakamura Y., Nagaya T., Choyke P.L., Hasegawa Y., Kobayashi H. (2016). Spatially selective depletion of tumor-associated regulatory T cells with near-infrared photoimmunotherapy. Sci. Transl. Med..

[26] Syn N.L., Teng M.W., Mok T.S., Soo R.A. (2017). De-novo and acquired resistance to immune checkpoint targeting. Lancet Oncol..

[27] Ling S.P., Ming L.C., Dhaliwal J.S., Gupta M., Ardianto C., Goh K.W., Hussain Z., Shafqat N. (2022). Role of Immunotherapy in the Treatment of Cancer: A Systematic Review. Cancers.

[28] Wang X., Teng F., Kong L., Yu J. (2016). PD-L1 expression in human cancers and its association with clinical outcomes. Onco Targets Ther..

[29] Fan Y., Zhang C., Jin S., Gao Z., Cao J., Wang A., Li D., Wang Q., Sun X., Bai D. (2019). Progress of immune checkpoint therapy in the clinic (Review). Oncol. Rep..

[30] Liu X., Guo C.Y., Tou F.F., Wen X.M., Kuang Y.K., Zhu Q., Hu H. (2020). Association of PD-L1 expression status with the efficacy of PD-1/PD-L1 inhibitors and overall survival in solid tumours: A systematic review and meta-analysis. Int. J. Cancer.

[31] Sahu M., Suryawanshi H. (2021). Immunotherapy: The future of cancer treatment. J. Oral Maxillofac. Pathol..

[32] The American Association for Cancer Research (AACR) Cancer Progress Report 2023. Spotlight on Immunotherapy: Pushing the Frontier of Cancer Medicine.

[33] Yervoy FDA Approval History. https://www.drugs.com/history/yervoy.html.

[34] Tremelimumab. DrugBank. https://go.drugbank.com/drugs/DB11771.

[35] Pembrolizumab. DrugBank. https://go.drugbank.com/drugs/DB09037.

[36] KEGG DRUG Database. DRUG: Nivolumab. https://www.kegg.jp/entry/D10316.

[37] Cemiplimab. DrugBank. https://go.drugbank.com/drugs/DB14707.

[38] Dostarlimab. DrugBank. https://go.drugbank.com/drugs/DB15627.

[39] Retifanlimab. DrugBank. https://go.drugbank.com/drugs/DB15766.

[40] Toripalimab. DrugBank. https://go.drugbank.com/drugs/DB15043.

[41] KEGG DRUG Database. DRUG: Toripalimab. https://www.kegg.jp/entry/D12202.

[42] TEVIMBRA Approved in U.S. for First-Line Treatment of Advanced Esophageal Squamous Cell Carcinoma in Combination with Chemotherapy. BeiGene, Ltd. https://ir.beigene.com/news/tevimbra-approved-in-u-s-for-first-line-treatment-of-advanced-esophageal-squamous-cell-carcinoma-in-combination/8379a7c3-35ce-45af-82d3-164c64ecf37c/.

[43] DRUG: Tislelizumab. KEGG. https://www.kegg.jp/entry/D11487.

[44] Zhang L., Geng Z., Hao B., Geng Q. (2022). Tislelizumab: A Modified Anti-tumor Programmed Death Receptor 1 Antibody. Cancer Control..

[45] Atezolizumab. DrugBank. https://go.drugbank.com/drugs/DB11595.

[46] DRUG: Atezolizumab. KEGG. https://www.kegg.jp/entry/D10773.

[47] Avelumab. DrugBank. https://go.drugbank.com/drugs/DB11945.

[48] Durvalumab. DrugBank. https://go.drugbank.com/drugs/DB11714.

[49] FDA Approves Cosibelimab-Ipdl for Metastatic or Locally Advanced Cutaneous Squamous Cell Carcinoma. U.S. Food and Drug Administration (FDA). https://www.fda.gov/drugs/resources-information-approved-drugs/fda-approves-cosibelimab-ipdl-metastatic-or-locally-advanced-cutaneous-squamous-cell-carcinoma.

[50] Cosibelimab. Wikipedia, the Free Encyclopedia. https://en.wikipedia.org/wiki/Cosibelimab.

[51] DRUG: Nivolumab and Relatlimab. KEGG. https://www.kegg.jp/entry/D12334.

[52] Nivolumab/Relatlimab. Wikipedia, the Free Encyclopedia. https://en.wikipedia.org/wiki/Nivolumab/relatlimab.

[53] Opdualag Becomes First FDA-Approved Immunotherapy to Target LAG-3. National Cancer Institute (NCI). 6 April 2022. https://www.cancer.gov/news-events/cancer-currents-blog/2022/fda-opdualag-melanoma-lag-3.

[54] (2023). Product Monograph Including Patient Medication Information. ^Pr^ Opdualag^TM^ Nivolumab and Relatlimab for Injection. Bristol-Myers Squibb Canada Montreal, Canada, SEP 13. https://www.bms.com/assets/bms/ca/documents/productmonograph/OPDUALAG_EN_PM.pdf.

[55] Lu C., Tan Y. (2024). Promising Immunotherapy Targets: TIM3, LAG3, and TIGIT Joined the Party. Mol. Ther. Oncol..

[56] Wang Z., Wu X. (2020). Study and analysis of antitumor resistance mechanism of PD1/PD-L1 immune checkpoint blocker. Cancer Med..

[57] Naimi A., Mohammed R.N., Raji A., Chupradit S., Yumashev A.V., Suksatan W., Shalaby M.N., Thangavelu L., Kamrava S., Shomali N. (2022). Tumor immunotherapies by immune checkpoint inhibitors (ICIs); the pros and cons. Cell Commun. Signal..

[58] Yan T., Yu L., Shangguan D., Li W., Liu N., Chen Y., Fu Y., Tang J., Liao D. (2023). Advances in pharmacokinetics and pharmacodynamics of PD-1/PD-L1 inhibitors. Int. Immunopharmacol..

[59] Marei H.E., Hasan A., Pozzoli G., Cenciarelli C. (2023). Cancer immunotherapy with immune checkpoint inhibitors (ICIs): Potential, mechanisms of resistance, and strategies for reinvigorating T cell responsiveness when resistance is acquired. Cancer Cell Int..

[60] Zhuang H., Cheng L., Wang Y., Zhang Y.K., Zhao M.F., Liang G.D., Zhang M.C., Li Y.G., Zhao J.B., Gao Y.N. (2019). Dysbiosis of the Gut Microbiome in Lung Cancer. Front. Cell Infect. Microbiol..

[61] Ferreira de Oliveira J.M.P., Santos C., Fernandes E. (2020). Therapeutic potential of hesperidin and its aglycone hesperetin: Cell cycle regulation and apoptosis induction in cancer models. Phytomedicine.

[62] Luo L., Lin C., Wang P., Cao D., Lin Y., Wang W., Zhao Y., Shi Y., Gao Z., Kang X. (2023). Combined Use of Immune Checkpoint Inhibitors and Phytochemicals as a Novel Therapeutic Strategy against Cancer. J. Cancer.

[63] Roszkowski S. (2023). Application of Polyphenols and Flavonoids in Oncological Therapy. Molecules.

[64] Vareed S.K., Kakarala M., Ruffin M.T., Crowell J.A., Normolle D.P., Djuric Z., Brenner D.E. (2008). Pharmacokinetics of curcumin conjugate metabolites in healthy human subjects. Cancer Epidemiol. Biomarkers Prev..

[65] Huang M., Zhai B.T., Fan Y., Sun J., Shi Y.J., Zhang X.F., Zou J.B., Wang J.W., Guo D.Y. (2023). Targeted Drug Delivery Systems for Curcumin in Breast Cancer Therapy. Int. J. Nanomed..

[66] Lucas J., Hsieh T.C., Halicka H.D., Darzynkiewicz Z., Wu J.M. (2018). Upregulation of PD-L1 expression by resveratrol and piceatannol in breast and colorectal cancer cells occurs via HDAC3/p300-mediated NF-κB signaling. Int. J. Oncol..

[67] Bonaventura P., Shekarian T., Alcazer V., Valladeau-Guilemond J., Valsesia-Wittmann S., Amigorena S., Caux C., Depil S. (2019). Cold Tumors: A Therapeutic Challenge for Immunotherapy. Front. Immunol..

[68] Dubbs S.B. (2018). The Latest Cancer Agents and Their Complications. Emerg. Med. Clin. N. Am..

[69] Maruoka Y., Nagaya T., Nakamura Y., Sato K., Ogata F., Okuyama S., Choyke P.L., Kobayashi H. (2017). Evaluation of Early Therapeutic Effects after Near-Infrared Photoimmunotherapy (NIR-PIT) Using Luciferase-Luciferin Photon-Counting and Fluorescence Imaging. Mol. Pharm..

[70] Kobayashi H., Choyke P.L. (2019). Near-Infrared Photoimmunotherapy of Cancer. Acc. Chem. Res..

[71] Wakiyama H., Kato T., Furusawa A., Choyke P.L., Kobayashi H. (2021). Near infrared photoimmunotherapy of cancer; possible clinical applications. Nanophotonics.

[72] Furusawa A., Choyke P.L., Kobayashi H. (2022). NIR-PIT: Will it become a standard cancer treatment?. Front. Oncol..

[73] Cetuximab Sarotalocan|ASP-1929|RM-1929 ADC Review. J. Antib.-Drug Conjugates..

[74] Kobayashi H., Choyke P.L. (2024). The role of interventional radiology and molecular imaging for near infrared photoimmunotherapy. Jpn. J. Radiol..

[75] Nakamura Y., Ohler Z.W., Householder D., Nagaya T., Sato K., Okuyama S., Ogata F., Daar D., Hoa T., Choyke P.L. (2017). Near Infrared Photoimmunotherapy in a Transgenic Mouse Model of Spontaneous Epidermal Growth Factor Receptor (EGFR)-expressing Lung Cancer. Mol. Cancer Ther..

[76] Miyazaki N.L., Furusawa A., Choyke P.L., Kobayashi H. (2023). Review of RM-1929 Near-Infrared Photoimmunotherapy Clinical Efficacy for Unresectable and/or Recurrent Head and Neck Squamous Cell Carcinoma. Cancers.

[77] Rakuten Medical Press Releases Rakuten Medical Announces First Patient Treatment in India in Global Phase 3 Trial of Alluminox Treatment (Photoimmunotherapy) Using ASP-1929 for Recurrent Head and Neck Cancer. 22 January 2024. https://rakuten-med.com/us/news/press-releases/2024/01/22/7788/.

[78] Ailioaie L.M., Ailioaie C., Litscher G. (2023). Synergistic Nanomedicine: Photodynamic, Photothermal and Photoimmune Therapy in Hepatocellular Carcinoma: Fulfilling the Myth of Prometheus?. Int. J. Mol. Sci..

[79] Cui Y., Xu Y., Li Y., Sun Y., Hu J., Jia J., Li X. (2023). Antibody Drug Conjugates of Near-Infrared Photoimmunotherapy (NIR-PIT) in Breast Cancers. Technol. Cancer Res. Treat..

[80] Monaco H., Yokomizo S., Choi H.S., Kashiwagi S. (2022). Quickly evolving near-infrared photoimmunotherapy provides multifaceted approach to modern cancer treatment. View.

[81] Sano K., Nakajima T., Choyke P.L., Kobayashi H. (2014). The effect of photoimmunotherapy followed by liposomal daunorubicin in a mixed tumor model: A demonstration of the super-enhanced permeability and retention effect after photoimmunotherapy. Molecular cancer therapeutics. Mol. Cancer Ther..

[82] Kobayashi H., Choyke P.L. (2016). Super enhanced permeability and retention (SUPR) effects in tumors following near infrared photoimmunotherapy. Nanoscale.

[83] Wei D., Qi J., Hamblin M.R., Wen X., Jiang X., Yang H. (2022). Near-infrared photoimmunotherapy: Design and potential applications for cancer treatment and beyond. Theranostics.

[84] Okada R., Furusawa A., Inagaki F., Wakiyama H., Kato T., Okuyama S., Furumoto H., Fukushima H., Choyke P.L., Kobayashi H. (2021). Endoscopic near-infrared photoimmunotherapy in an orthotopic head and neck cancer model. Cancer Sci..

[85] Kato T., Wakiyama H., Furusawa A., Choyke P.L., Kobayashi H. (2022). Near Infrared Photoimmunotherapy; A Review of Targets for Cancer Therapy. Cancers.

[86] Sung H., Ferlay J., Siegel R.L., Laversanne M., Soerjomataram I., Jemal A., Bray F. (2021). Global cancer statistics 2020: GLOBOCAN estimates of incidence and mortality worldwide for 36 cancers in 185 countries. CA Cancer J. Clin..

[87] Barsouk A., Aluru J.S., Rawla P., Saginala K., Barsouk A. (2023). Epidemiology, Risk Factors, and Prevention of Head and Neck Squamous Cell Carcinoma. Med. Sci..

[88] Bray F., Laversanne M., Sung H., Ferlay J., Siegel R.L., Soerjomataram I., Jemal A. (2024). Global cancer statistics 2022: GLOBOCAN estimates of incidence and mortality worldwide for 36 cancers in 185 countries. CA Cancer J. Clin..

[89] Gormley M., Creaney G., Schache A., Ingarfield K., Conway D.I. (2022). Reviewing the epidemiology of head and neck cancer: Definitions, trends and risk factors. Br. Dent. J..

[90] Lim I., Tan J., Alam A., Idrees M., Brenan P.A., Coletta R.D., Kujan O. (2024). Epigenetics in the diagnosis and prognosis of head and neck cancer: A systematic review. J. Oral. Pathol. Med..

[91] Chow L.Q.M. (2020). Head and Neck Cancer. N. Engl. J. Med..

[92] Liu Y., Zhang N., Wen Y., Wen J. (2024). Head and neck cancer: Pathogenesis and targeted therapy. MedComm.

[93] EpiCast Report: Head and Neck Cancers—Epidemiology Forecast to 2024. https://www.globaldata.com/store/report/epicast-report-head-and-neck-cancers-epidemiology-forecast-to-2024/.

[94] Lee B. Head and Neck Cancer Treatment & Pharmacologic Management. *Cancer Therapy Advisor*, 9 May 2023.

[95] Rades D., Zwaan I., Soror T., Idel C., Pries R., Bruchhage K.L., Hakim S.G., Yu N.Y. (2023). Chemoradiation with Cisplatin vs. Carboplatin for Squamous Cell Carcinoma of the Head and Neck (SCCHN). Cancers.

[96] Study of RM-1929 and Photoimmunotherapy in Patients with Recurrent Head and Neck Cancer. ClinicalTrials.gov ID: NCT02422979. NCT02422979.

[97] Kochuparambil S.T., McDonald D., Fidler M., Stenson K., Vasan N., Razaq A.M. (2017). A phase 1, multicenter, open-label, dose-escalation, combination study of RM-1929 and photoimmunotherapy in patients with recurrent head and neck cancer. 1051PD Abstract Book of the 42nd ESMO Congress (ESMO 2017). Ann. Oncol..

[98] Gillenwater M.A., Cognetti D.M., Johnson J.M., Curry J., Kochuparambil S.T., McDonald D., Fidler M.J., Stenson K., Vasan N., Razaq M. (2018). 2018 ASCO Annual Meeting I, Abstract 6039. RM-1929 photo-immunotherapy in patients with recurrent head and neck cancer: Results of a multicenter phase 2a open-label clinical trial. J. Clin. Oncol..

[99] Cognetti D.M., Johnson J.M., Curry M.J., Mott F., Kochuparambil S.T., McDonald D., Fidler M.J., Stenson K., Vasan N.R., Razaq M. (2019). 2019 ASCO Annual Meeting I, Abstract 6014. Results of a phase 2a, multicenter, open-label, study of RM-1929 photoimmunotherapy (PIT) in patients with locoregional, recurrent head and neck squamous cell carcinoma (rHNSCC). J. Clin. Oncol..

[100] Cognetti D.M., Johnson J.M., Curry M.J., Kochuparambil S.T., McDonald D., Mott F., Fidler M.J., Stenson K., Vasan N.R., Razaq M.A. (2021). Phase 1/2a, open-label, multicenter study of RM-1929 photoimmunotherapy in patients with locoregional, recurrent head and neck squamous cell carcinoma. Head Neck.

[101] A Phase 3, Randomized, Double-Arm, Open-Label, Controlled Trial of ASP-1929 Photoimmunotherapy Versus Physician’s Choice Standard of Care for the Treatment of Locoregional, Recurrent Head and Neck Squamous Cell Carcinoma in Patients Who Have Failed or Progressed On or After at Least Two Lines of Therapy, of Which at Least One Line Must Be Systemic Therapy. ClinicalTrials.gov ID: NCT03769506. NCT03769506.

[102] An Open-Label Study Using ASP-1929 Photoimmunotherapy in Combination with Anti-PD1 Therapy in EGFR Expressing Advanced Solid Tumors. ClinicalTrials.gov ID: NCT04305795. NCT04305795.

[103] Cognetti D.M., Curry J.M., Civantos F.F., Valentino J., Agbaje-Williams M., Danesi H., Dong H., Larracas C., Veresh B., Gillenwater A.M. (2024). 2024 ASCO Annual Meeting I, Abstract 6083. Recent safety and efficacy findings from a phase 1b/2 open-label combination study of ASP-1929 photoimmunotherapy with anti-PD-1 therapy in EGFR-expressing advanced head and neck squamous cell carcinoma (HNSCC). J. Clin. Oncol..

[104] A Phase 1 First-in-Human, Drug-Dose Escalation Study of RM-1995 Photoimmunotherapy, as Monotherapy or Combined with Pembrolizumab, in Patients with Advanced Cutaneous Squamous Cell Carcinoma or with Head and Neck Squamous Cell Carcinoma. ClinicalTrials.gov ID: NCT05220748. NCT05220748.

[105] A Phase 2 Single-Arm Study of ASP-1929 Photoimmunotherapy Combined with Pembrolizumab in Patients with Locoregional Recurrent Squamous Cell Carcinoma of the Head and Neck, with or Without Metastases, Not Amenable to Curative Local Treatment. ClinicalTrials.gov ID: NCT05265013. NCT05265013.

[106] Phase 2, Open-Label, Single-Arm, Window of Opportunity Study of ASP-1929 Photoimmunotherapy with Fluorescence Imaging in Patients with Operable Primary or Recurrent Head and Neck or Cutaneous Squamous Cell Carcinoma. ClinicalTrials.gov ID: NCT05182866. NCT05182866.

[107] Maruoka Y., Wakiyama H., Choyke P.L., Kobayashi H. (2021). Near infrared photoimmunotherapy for cancers: A translational perspective. EBioMedicine.

[108] Peng Z., Wang Y., Fan R., Gao K., Xie S., Wang F., Zhang J., Zhang H., He Y., Xie Z. (2022). Treatment of Recurrent Nasopharyngeal Carcinoma: A Sequential Challenge. Cancers.

[109] Tahara M., Okano S., Enokida T., Ueda Y., Fujisawa T., Shinozaki T., Tomioka T., Okano W., Biel M.A., Ishida K. (2021). A phase I, single-center, open-label study of RM-1929 photoimmunotherapy in Japanese patients with recurrent head and neck squamous cell carcinoma. Int. J. Clin. Oncol..

[110] Kushihashi Y., Masubuchi T., Okamoto I., Fushimi C., Hanyu K., Yamauchi M., Tada Y., Miura K. (2022). Photoimmunotherapy for Local Recurrence of Nasopharyngeal Carcinoma: A Case Report. Int. J. Otorhinolaryngol. Head Neck Surg..

[111] Kroemer G., Galluzzi L., Kepp O., Zitvogel L. (2013). Immunogenic cell death in cancer therapy. Annu. Rev. Immunol..

[112] Dudek A.M., Garg A.D., Krysko D.V., De Ruysscher D., Agostinis P. (2013). Inducers of immunogenic cancer cell death. Cytokine Growth Factor. Rev..

[113] Ogawa M., Tomita Y., Nakamura Y., Lee M.J., Lee S., Tomita S., Nagaya T., Sato K., Yamauchi T., Iwai H. (2017). Immunogenic cancer cell death selectively induced by near infrared photoimmunotherapy initiates host tumor immunity. Oncotarget.

[114] Nishikawa D., Suzuki H., Beppu S., Terada H., Sawabe M., Hanai N. (2022). Near-Infrared Photoimmunotherapy for Oropharyngeal Cancer. Cancers.

[115] Sato K., Ando K., Okuyama S., Moriguchi S., Ogura T., Totoki S., Hanaoka H., Nagaya T., Kokawa R., Takakura H. (2018). Photoinduced Ligand Release from a Silicon Phthalocyanine Dye Conjugated with Monoclonal Antibodies: A Mechanism of Cancer Cell Cytotoxicity after Near-Infrared Photoimmunotherapy. ACS Cent. Sci..

[116] Okamoto I., Okada T., Tokashiki K., Tsukahara K. (2022). Photoimmunotherapy for Managing Recurrent Laryngeal Cancer Cervical Lesions: A Case Report. Case Rep. Oncol..

[117] Okamoto I., Okada T., Tokashiki K., Tsukahara K. (2022). A Case Treated with Photoimmunotherapy Under a Navigation System for Recurrent Lesions of the Lateral Pterygoid Muscle. In Vivo.

[118] Okamoto I., Okada T., Tokashiki K., Tsukahara K. (2022). Quality-of-Life Evaluation of Patients with Unresectable Locally Advanced or Locally Recurrent Head and Neck Carcinoma Treated with Head and Neck Photoimmunotherapy. Cancers.

[119] Idogawa H., Shinozaki T., Okano W., Matsuura K., Hayashi R. (2023). Nasopharyngeal Carcinoma Treated with Photoimmunotherapy. Cureus.

[120] Beyer W. (1996). Systems for light application and dosimetry in photodynamic therapy. J. Photochem. Photobiol. B.

[121] Vesselov L., Whittington W., Lilge L. (2005). Design and performance of thin cylindrical diffusers created in Ge-doped multimode optical fibers. Appl. Opt..

[122] Shafirstein G., Bellnier D., Oakley E., Hamilton S., Potasek M., Beeson K., Parilov E. (2017). Interstitial Photodynamic Therapy—A Focused Review. Cancers.

[123] Stepp H., Sroka R. (2024). Simple Characterization of Cylindrical Diffuser Fibers with a Fluorescent Layer. Lasers Surg. Med..

[124] Ta M.D., Kim Y., Shin H., Truong V.G., Kang H.W. (2024). Quantitative investigations on light emission profiles for interstitial laser treatment. Biomed. Opt. Express.

[125] Koyama S., Ehara H., Donishi R., Morisaki T., Ogura T., Taira K., Fukuhara T., Fujiwara K. (2023). Photoimmunotherapy with surgical navigation and computed tomography guidance for recurrent maxillary sinus carcinoma. Auris Nasus Larynx.

[126] Omura G., Honma Y., Matsumoto Y., Shinozaki T., Itoyama M., Eguchi K., Sakai T., Yokoyama K., Watanabe T., Ohara A. (2023). Transnasal photoimmunotherapy with cetuximab sarotalocan sodium: Outcomes on the local recurrence of nasopharyngeal squamous cell carcinoma. Auris Nasus Larynx.

[127] Shibutani Y., Sato H., Suzuki S., Shinozaki T., Kamata H., Sugisaki K., Kawanobe A., Uozumi S., Kawasaki T., Hayashi R. (2023). A Case Series on Pain Accompanying Photoimmunotherapy for Head and Neck Cancer. Healthcare.

[128] Shinozaki T., Matsuura K., Okano W., Tomioka T., Nishiya Y., Machida M., Hayashi R. (2023). Eligibility for Photoimmunotherapy in Patients with Unresectable Advanced or Recurrent Head and Neck Cancer and Changes before and after Systemic Therapy. Cancers.

[129] Moriya T., Hashimoto M., Matsushita H., Masuyama S., Yoshida R., Okada R., Furusawa A., Fujimura D., Wakiyama H., Kato T. (2022). Near-infrared photoimmunotherapy induced tumor cell death enhances tumor dendritic cell migration. Cancer Immunol. Immunother..

[130] Ishihara H., Nishikawa D., Muraoka D., Masago K., Beppu S., Terada H., Matsushita H., Hanai N. (2024). Changes in serum DAMPs and cytokines/chemokines during near-infrared photoimmunotherapy for patients with head and neck cancer. Cancer Med..

[131] Hanyu K., Okamoto I., Tokashiki K., Tsukahara K. (2024). A Case of Successful Treatment with an Immune Checkpoint Inhibitor after Head and Neck Photoimmunotherapy. Case Rep. Oncol..

[132] Hirakawa H., Ikegami T., Kinjyo H., Hayashi Y., Agena S., Higa T., Kondo S., Toyama M., Maeda H., Suzuki M. (2024). Feasibility of Near-infrared Photoimmunotherapy Combined with Immune Checkpoint Inhibitor Therapy in Unresectable Head and Neck Cancer. Anticancer. Res..

[133] Harrington K.J., Burtness B., Greil R., Soulières D., Tahara M., de Castro G., Psyrri A., Brana I., Basté N., Neupane P. (2023). Pembrolizumab with or without Chemotherapy in Recurrent or Metastatic Head and Neck Squamous Cell Carcinoma: Updated Results of the Phase III KEYNOTE-048 Study. J. Clin. Oncol..

[134] Oridate N., Takahashi S., Tanaka K., Shimizu Y., Fujimoto Y., Matsumoto K., Yokota T., Yamazaki T., Takahashi M., Ueda T. (2024). First-line pembrolizumab with or without chemotherapy for recurrent or metastatic head and neck squamous cell carcinoma: 5-year follow-up of the Japanese population of KEYNOTE-048. Int. J. Clin. Oncol..

[135] Kobayashi H., Furusawa A., Rosenberg A., Choyke P.L. (2021). Near-infrared photoimmunotherapy of cancer: A new approach that kills cancer cells and enhances anti-cancer host immunity. Int. Immunol..

[136] Hsu M.A., Okamura S.M., De Magalhaes Filho C.D., Bergeron D.M., Rodriguez A., West M., Yadav D., Heim R., Fong J.J., Garcia-Guzman M. (2023). Cancer-targeted photoimmunotherapy induces anti-tumor immunity and can be augmented by anti-PD-1 therapy for durable anticancer responses in an immunologically active murine tumor model. Cancer Immunol. Immunother..

[137] Koyama S., Ehara H., Donishi R., Taira K., Fukuhara T., Fujiwara K. (2024). Therapeutic Host Anticancer Immune Response through Photoimmunotherapy for Head and Neck Cancer May Overcome Resistance to Immune Checkpoint Inhibitors. Case Rep. Oncol..

[138] Okamoto I., Okada T., Tokashiki K., Tsukahara K. (2024). Two Cases of Emergency Tracheostomy After Head and Neck Photoimmunotherapy. Cancer Diagn. Progn..

[139] Kásler M., Fodor J., Oberna F., Major T., Polgár C., Takácsi-Nagy Z. (2008). Salvage surgery for locoregional failure after definitive radiotherapy for base of tongue cancer. In Vivo.

[140] Akyildiz A., Guven D.C., Koksal B., Karaoglan B.B., Kivrak D., Ismayilov R., Aslan F., Sutcuoglu O., Yazici O., Kadioglu A. (2024). Real-world evaluation of nivolumab in patients with non-nasopharyngeal recurrent or metastatic head and neck cancer: A retrospective multi-center study by the Turkish Oncology Group (TOG). Eur. Arch. Otorhinolaryngol..

[141] Tamagawa S., Okuda K., Nishikawa D., Kono M., Hotomi M. (2024). Near Infrared Photoimmunotherapy for Locoregionally Recurrent Oropharyngeal Squamous Cell Carcinoma at Tongue Base After Chemoradiotherapy: A Case Report and Literature Review. Cureus.

[142] Li W., Zhang H., Lu H., Wang H., Gu Y., Li H., Sun X., Yu H., Wang D. (2021). Clinical Outcomes of Salvage Endoscopic Nasopharyngectomy for Patients with Advanced Recurrent Nasopharyngeal Carcinoma. Front. Oncol..

[143] Lam W.K.J., Chan J.Y.K. (2018). Recent advances in the management of nasopharyngeal carcinoma. F1000Research.

[144] Ma H., Fang J., Zhong Q., Hou L., Feng L., He S., Wang R., Yang Y. (2022). Reconstruction of nasopharyngeal defect with submental flap during surgery for nasopharyngeal malignant tumors. Front. Surg..

[145] Kushihashi Y., Masubuchi T., Okamoto I., Fushimi C., Yamazaki M., Asano H., Aoki R., Fujii S., Asako Y., Tada Y. (2024). A Case of Photoimmunotherapy for Nasopharyngeal Carcinoma Requiring Emergency Tracheostomy. Case Rep. Oncol..

[146] Krüger-Genge A., Blocki A., Franke R.P., Jung F. (2019). Vascular Endothelial Cell Biology: An Update. Int. J. Mol. Sci..

[147] Wang S., Zhang Y. (2020). HMGB1 in inflammation and cancer. J. Hematol. Oncol..

[148] Teixeira G., Faria R. (2021). Inflammatory Mediators Leading to Edema Formation through Plasma Membrane Receptors. Infections and Sepsis Development.

[149] Caudell T.P., Mizell D.M. Augmented reality: An application of heads-up display technology to manual manufacturing processes. Proceedings of the Twenty-Fifth Hawaii International Conference on System Sciences.

[150] Lee K. (2012). Augmented Reality in Education and Training. TechTrends.

[151] Thomas D.J. (2016). Augmented reality in surgery: The Computer-Aided Medicine revolution. Int. J. Surg..

[152] History Of Virtual Reality. https://www.vrs.org.uk/virtual-reality/history.html.

[153] Thomas Caudell. Coined the Term “Augmented Reality” at Boeing in 1990. https://www.awexr.com/hall-of-fame/20-thomas-caudell.

[154] History of Augmented Reality: The Timeline. GlobalData Thematic Research. https://www.verdict.co.uk/augmented-reality-timeline/?cf-view.

[155] Augmented Reality. Wikipedia, the Free Encyclopedia. https://en.wikipedia.org/wiki/Augmented_reality.

[156] Okada R., Ito T., Kawabe H., Tsutsumi T., Asakage T. (2024). Mixed reality-supported near-infrared photoimmunotherapy for oropharyngeal cancer: A case report. Ann. Med. Surg..

[157] Okamoto I. (2025). Photoimmunotherapy for head and neck cancer: A systematic review. Auris Nasus Larynx.

[158] Walkty A., Embil J. (2019). Lemierre’s Syndrome. N. Engl. J. Med..

[159] Le C., Gennaro D., Marshall D., Alaev O., Bryan A., Gelfman A., Wang Z. (2019). Lemierre’s syndrome: One rare disease-Two case studies. J. Clin. Pharm. Ther..

[160] García Romero J.M., Jaime Vargas F.P., Bravo Quiroz J.G., Pérez Mondragón D., Hernández Guillen P. (2024). Lemierre’s Syndrome: A Case Report of Thrombophlebitis of the Internal Jugular Vein Induced by Laryngeal Cancer. Cureus.

[161] Allen B.W., Anjum F., Bentley T.P. (2025). Lemierre Syndrome. [Updated 2023 Jul 31]. StatPearls [Internet].

[162] Nishimura M., Okamoto I., Ito T., Tokashiki K., Tsukahara K. (2024). Lemierre’s Syndrome after Head and Neck Photoimmunotherapy for Local Recurrence of Nasopharyngeal Carcinoma. Case Rep. Oncol..

[163] Hahn J., Nordmann-Kleiner M., Hoffmann T.K., Greve J. (2019). Thrombosis of the internal jugular vein in the ENT-department–Prevalence, causes and therapy: A retrospective analysis. Auris Nasus Larynx.

[164] Pan Y., Shi Z., Ye B., Da Q., Wang C., Shen Y., Xiang M. (2024). Surgical intervention of Lemierre’s syndrome: A case report and review of the literature. J. Med. Case Rep..

